# Arbeitsbelastung und psychische Gesundheit von Pflegekräften in Deutschland während der COVID-19-Pandemie – Ein Scoping-Review

**DOI:** 10.1007/s00103-024-03984-5

**Published:** 2024-12-03

**Authors:** Dagmar Arndt, Thomas Hering

**Affiliations:** 1 Fachbereich Soziale Arbeit, Gesundheit und Medien, Hochschule Magdeburg-Stendal, Breitscheidstraße 2, 39114 Magdeburg, Deutschland; 2 Fachbereich Angewandte Humanwissenschaften, Hochschule Magdeburg-Stendal, Stendal, Deutschland

**Keywords:** Pflegefachkräfte, Pandemie, Deutschland, Arbeitsbelastung, Psychische Gesundheit, Nursing staff, Pandemic, Germany, Job demands, Mental health

## Abstract

**Hintergrund:**

Im März 2023 existierte für Deutschland keine zusammenfassende Übersichtarbeit zu Arbeitsbelastung, Ressourcen, Beanspruchungsfolgen in der COVID-19-Pandemie für Pflegefachkräfte. Vor dem Hintergrund länderspezifischer Unterschiede hinsichtlich Pandemieverlauf/-management und Organisation des Gesundheitswesens soll eine gesonderte Betrachtung von Daten aus Deutschland erfolgen.

**Methode:**

Eine systematische Datenbankrecherche (PubMed/Medline, PsycINFO) brachte 50 relevante Studien, die Eingang in das Scoping-Review fanden.

**Ergebnisse:**

Die Studien basieren auf Querschnittdesigns, stammen zumeist aus dem Setting Klinik und dem ersten Pandemiejahr. Häufig untersuchte Belastungsfaktoren waren Arbeitsintensivierung, fehlende Schutzausrüstung, schnell wechselnde Regelungen, Kontakt zu COVID-19-Patienten, pandemiebezogene Sorgen/Ängste. Pandemiephasen- und settingspezifische Belastungskaskaden wurden deutlich. Häufig untersuchte psychische Beanspruchungen (u. a. Stresserleben, Angst, Depressivität, Burn-out, Berufs‑/Arbeitsplatzwechselabsichten) waren in Gruppen mit ungünstigeren Belastungswerten höher. Pflegende berichteten zumeist ungünstigere Merkmalsausprägungen als Mediziner. Soziale Unterstützung, Belohnung/Wertschätzung, Humor, Resilienz und Kohärenzgefühl erwiesen sich als Ressourcen.

**Schlussfolgerung:**

In Vorbereitung auf weitere Pandemien und Krisen sollten präpandemisch vorliegende Belastungsfaktoren durch gezielte Maßnahmen abgebaut und relevante Ressourcen gestärkt werden. Pandemiespezifische Belastungsfaktoren sollten systematisch reflektiert, organisationsübergreifend Lösungen erarbeitet werden. Längsschnittstudien unter Nutzung validierter Instrumente sowie Interventionsstudien sollten zukünftig in den Fokus der Forschungsförderung fallen.

**Zusatzmaterial online:**

Zusätzliche Informationen sind in der Online-Version dieses Artikels (10.1007/s00103-024-03984-5) enthalten.

## Abstract (en)

**Background:**

In March 2023, there was no comprehensive overview of job demands, resources, and mental health consequences for nurses in Germany during the COVID-19 pandemic. Because of country-specific differences with regard to the course/management of the pandemic and the organization of the healthcare system, data from Germany were analyzed specifically.

**Methods:**

A systematic search in databases (PubMed/Medline, PsycINFO) yielded 50 relevant studies that were included in the scoping review.

**Results:**

The studies were based on cross-sectional designs, mostly from the hospital setting and the first year of the pandemic. Frequently investigated job demands were work intensification, lack of protective equipment, rapidly changing regulations, contact with COVID-19 patients, and pandemic-related worries/anxieties. Pandemic phase and setting-specific demand cascades became clear. Frequently examined mental health problems (including stress experience, anxiety, depression, burnout, and intention to change career/job) were higher in groups with higher levels of reported job demands. Nurses generally reported less favorable values than medical professionals. Social support, reward/appreciation, humor, resilience, and sense of coherence proved to be resources.

**Conclusion:**

In preparation for further pandemics and crises, pre-pandemic job demands should be reduced through targeted measures and relevant resources should be strengthened. Pandemic-specific job demands should be systematically reflected and cross-organizational solutions developed. Longitudinal studies using validated instruments and intervention studies should be the focus of future research funding.

## Hintergrund

Seit Beginn der COVID-19-Pandemie wurden zahlreiche Studien und Übersichtarbeiten zu Arbeitsbelastung, Ressourcen und Beanspruchungsfolgen (vgl. DIN EN ISO 10075‑1^1^ [1]) bei Pflegefachkräften und Medizinern^2^ durchgeführt. Nationale sowie professionsspezifische Besonderheiten wurden seltener fokussiert betrachtet (u. a. [2–4]).

Zukünftig sind Krisenereignisse nicht auszuschließen und es gilt, „aus den Erfahrungen zeitnah Konsequenzen für die Vorbereitung auf zukünftige Krisen abzuleiten“ [5]. Dazu zählt auch die Analyse der psychosozialen Arbeitsbelastung, psychischer Beeinträchtigungen, Ressourcen und Bewältigungsstrategien, die Basis für die Entwicklung von verhaltens- und verhältnispräventiven Angeboten in Gesundheitseinrichtungen und von Entscheidungen zur Gestaltung des Gesundheitssystems sein sollte. Denn die Pandemie traf in Deutschland auf ein durch Fachkräftemangel und Arbeitsverdichtung gekennzeichnetes System [6, 7]. Vor diesem Hintergrund sowie aufgrund länderspezifischer Unterschiede im Pandemieverlauf/-management und der Organisation des Gesundheitswesens gewinnt die systematische Zusammenfassung nationaler Studien an Bedeutung.

Im Rahmen eines Scoping-Reviews soll der Forschungstand zur psychosozialen Arbeitsbelastung in der Pandemie, zu arbeits- und personbezogenen Ressourcen sowie Beanspruchungsfolgen beim Personal im deutschen Gesundheitswesen untersucht werden. (Arbeits‑)Belastung meint die Gesamtheit der Belastungsfaktoren, die psychisch auf Mitarbeitende einwirken. Die Begriffe Beanspruchung bzw. Beanspruchungsfolgen werden für die unmittelbaren bzw. langfristigen Auswirkungen der Belastung bei Mitarbeitenden verwendet. Es interessieren auch Veränderungen in der Pandemie bzw. im Vergleich zur präpandemischen Phase sowie Zusammenhänge zwischen Arbeitsbelastung, psychischer(n) Beanspruchung(sfolgen), Ressourcen, Bewältigungsstrategien. Aufgrund der Verfügbarkeit einer ausreichenden Zahl an Studien liegt der Fokus auf Pflegefachpersonal aus unterschiedlichen Versorgungsbereichen, u. a. Kliniken, stationären Langzeitpflege- und ambulanten Pflegeeinrichtungen. Theoretische Basis der Untersuchung sind empirisch geprüfte (arbeits‑)psychologische Modellannahmen, wonach die Bewertung von Belastungsfaktoren, Ressourcen und Bewältigungsmöglichkeiten bedeutsame Einflussfaktoren für die Ausbildung von Beanspruchungsfolgen sind [8, 9].

## Methode

Die Analyse basiert auf einem Scoping-Review [10]. Die Recherche wurde von Juni 2023 bis Februar 2024 in Medline/PubMed und PsycINFO durchgeführt. Ergänzend erfolgte eine Handsuche in Archiven von Fachzeitschriften, Referenzlisten der Treffer, Websites. Suchbegriffe wurden in Anlehnung an vorliegende internationale Reviews und auf Basis einer Schlagwortrecherche in den genutzten Datenbanken formuliert (vgl. Onlinematerial, Tabelle Z1).

Eingeschlossen wurden Studien, deren Stichproben mindestens 75 % Pflegefachpersonen umfassten oder die Ergebnisse professionsspezifisch berichteten. Internationale Studien wurden berücksichtigt, wenn eine Differenzierung der Ergebnisse bezüglich des Berufes/Landes möglich war. Eingeschlossen wurden englisch- und deutschsprachige Studien. Im Vorfeld definierte Ausschlusskriterien bezogen sich auf die fehlende Ergebnisdifferenzierung nach Beruf/Land, einen fehlenden Bezug zur Fragestellung sowie auf nicht empirische Artikel einschließlich Reviews und Interventionsstudien. Die Bewertung der methodischen Qualität der Studien ist i. d. R. nicht Bestandteil eines Scoping-Reviews [10]. Dennoch wurden Minimalanforderungen formuliert: Veröffentlichung mit Peer-Review-Verfahren, nachvollziehbar beschriebener Einsatz von Messinstrumenten, Angaben zu und Einsatz geeigneter statistischer Verfahren, vollständiger Bericht inferenzstatistischer Ergebnisse (u. a. Prüfverfahren, Testgröße), Stichprobengrößen von *n* > 30 oder Überprüfung der Normalverteilungsannahme.

Im ersten Schritt erfolgte die Sichtung der gefundenen Studien durch 2 unabhängige Reviewende (DA und AG, siehe Danksagung). Doppelungen wurden ausgeschlossen, Titel und Abstracts der Treffer beurteilt und bei Fehlpassung Treffer ausgeschlossen. In der anschließenden Sichtung der Volltexte wurde bei Dissens im Reviewer-Team (DA, AG, TH) eine Entscheidung zur Aufnahme diskutiert. Weiter wurden grundlegende Anforderungen an die methodische Qualität der inhaltlich relevanten Studien durch 2 Reviewende (DA, TH) beurteilt.

800 Studien wurden mittels Datenbankrecherche, 6 Studien durch Handsuche identifiziert. 50 Studien wurden ins Review eingeschlossen (vgl. Onlinematerial, Abbildung Z1). Folgende Daten wurden extrahiert: Autoren, Publikationsjahr, Messzeitpunkt, Studiendesign, Stichprobenumfang, Setting, Geschlecht, Fragestellung, Hauptergebnisse.

## Ergebnisse

### Beschreibung der eingeschlossenen Studien

Entsprechend der Phaseneinteilung zur Beschreibung des COVID-19-Geschehens in Deutschland [11] können 22 Studien der ersten Welle (03–05/2020) und dem Sommerplateau (05–09/2020) zugeordnet werden. 12 Studien stammen aus Welle 2 (09/2020–02/2021), 8 Studien aus Welle 3 (03–06/2021), eine Studie aus Welle 5 (12/2021–05/2022), 2 Studien aus der Zeit nach Mai 2022. 4 wellenübergreifende Studien beziehen sich auf die Wellen 1–3. In einer Studie wurden keine Angaben zum Erhebungszeitraum gefunden (vgl. Onlinematerial, Tabellen Z2 und Z3).

5 Studien berühren den Bereich der ambulanten Pflege [12–16]. 9 Studien untersuchen gemischte Stichproben aus unterschiedlichen Settings [17–25], 11 Studien stammen aus dem Setting Pflegeheim [26–36]. 25 Studien wurden mehrheitlich mit Klinikpersonal durchgeführt (ca. 70 %; [37–61]). 36 Studien basieren auf quantitativen Designs, insbesondere Online-Befragungen, 14 Studien waren qualitativ (Interviewstudien). Die Studienergebnisse beruhen bis auf 2 Studien [40, 42] auf Querschnittdaten bzw. werden als solche berichtet, wenngleich einige Erhebungen zu unterschiedlichen Messzeitpunkten in gleichen Studiensettings durchgeführt wurden^3^ (vgl. Onlinematerial, Tabelle Z3). Der Stichprobenumfang variierte zwischen 55 und 2689 Teilnehmenden in quantitativen und 8 bis 510 Teilnehmenden in qualitativen Studien. Der Frauenanteil bewegte sich zwischen 61 % und 100 %. Da die Studien mehrheitlich auf Online-Befragungen basieren, sind Rücklaufquoten schwerer ermittelbar.

### Arbeitsbelastung in der Pandemie

Zu den am häufigsten untersuchten Belastungsfaktoren zählen Workload, Hygiene- und Informationsanforderungen, Qualifikationsanforderungen, emotionale Anforderungen (Tab. 1). Soziale Belastungsfaktoren ergaben sich aus der Kommunikation und Beziehung zwischen Fachkräften und Patienten/Angehörigen, innerhalb der Teams sowie zwischen Mitarbeitenden und Führungskräften, wenngleich Teamaspekte mehrheitlich als Ressourcen (z. B. Unterstützung, Zusammenhalt) diskutiert wurden. Die qualitativen Daten lassen auf enge Verknüpfungen der Belastungsfaktoren schließen, die entsprechend der Clusterung der Gemeinsamen Deutschen Arbeitsschutzstrategie (GDA) geordnet werden können [62].

**Tab. 1:** Belastungsfaktoren in der Pandemie entsprechend Clusterung der Gemeinsamen Deutschen Arbeitsschutzstrategie (GDA; [62])

Belastungsfaktorencluster	Belastungsfaktor	Ursachen und Erläuterung
Arbeitsorganisation und Arbeitszeit	Workload/Arbeitsintensivierung (inkl. Überstunden, fehlende Pausen; [14–17, 20, 21, 23, 24, 26, 27, 29–35, 38, 45, 48, 50, 51, 54–57, 60, 61])	*Personalmangel/-ausfall* (Quarantäne/Erkrankung, Kündigung, Impfpflicht)
		Aufwendige *Hygienemaßnahmen* (z. B. Testungen, Anlage Schutzausrüstung, Desinfektion)
		*Veränderte Alltagsroutinen* (u. a. Essensausgabe im Zimmer)
		*Übernahme pflegefachfremder Aufgaben* (z. B. Besuchergeschenke an der Pforte entgegennehmen, Videoanrufe für Bewohner/Angehörige, Anschaffung Tablets und Geräteeinweisung)
		Missverständnisse/*erhöhter Kommunikationsbedarf* (z. B. schriftliche Übergaben, Umsetzung wechselnder Regelungen)
		*Ausfall externer Dienste* (z. B. Friseur, ehrenamtliche Helfer) und der *Unterstützung durch Angehörige* (v. a. in Kliniken, Pflegeheimen)
		Dienstbesprechungen mit mehreren Kleinteams, Einbindung in Versorgung und Organisation, Planung- und Entscheidungsarbeit, Beschaffungsaufwand für Schutz- und Desinfektionsmaterial (*Führungskräfte*)
		Ergänzend: *Doppelbelastungen* durch Schließungen von Kitas/Schulen
	Erhöhter Kommunikationsbedarf [14, 15, 21, 26, 29, 34, 38]	Missverständnisse, z. B. durch schriftliche Übergaben
		Widersprüchliche, schnell wechselnde behördliche und innerorganisatorische Regelungen
Arbeitsmittel	Hygienemanagement [14–16, 21, 22, 26, 29, 33, 34, 38]	Mangel an Schutzausrüstung, Desinfektionsmitteln
		Neue, aufwendigere Hygienemaßnahmen, wechselnde Standards (s. oben)
Arbeitsinhalte/-aufgaben	Emotionale Anforderungen [12, 14, 15, 17–19, 21, 26, 27, 29, 32, 34–36, 38, 45, 46, 48, 50, 54–57, 60, 61]	*Pandemieassoziierte Sorgen/Ängste*: Sorge, sich selbst zu infizieren; Sorge, das Virus auf Familie/Angehörige, Patienten zu übertragen; um die eigene Gesundheit; um die Gesundheit von Familie, Patienten; generelle Bedrohungswahrnehmung; Zukunfts-/finanzielle Sorgen
		*Tod von Patienten*: Häufung von Todesfällen, Begleitung schwerer Verläufe, schwere Verläufe junger/gleichaltriger Patienten (v. a. Klinik, Pflegeheim)
		*Leid von Patienten*: Angst, Einsamkeit, Traurigkeit der Patienten, emotionale Begleitung durch Pflegende, Förderung sozialer Teilhabe (v. a. Pflegeheim, ambulante Pflege)
		*Diskriminierung/Stigmatisierung*: durch Freunde, Bekannte, Familie, Fremde, Vorgesetzte, Kollegen, Patienten; durch öffentliche Berichte; durch Distanzierung, Kontaktvermeidung, Ausgrenzung aufgrund von Angst vor Ansteckung; Unsicherheit; mangelndes Wissen; Impfskepsis; Schuldzuweisungen für Erkrankungsausbrüche
		*Moralische Belastung*: Einschränkung der Möglichkeiten für eine würdevolle Sterbebegleitung durch Kontaktbeschränkungen, Rationierung von Pflegeleistungen bis zur gefährlichen Pflege aufgrund Arbeitsverdichtung, Bedürfnis nach Sicherheit/Schutz und fehlende Schutzmaterialien, eingeschränkte medizinische Versorgung der Heime und Übernahme von medizinischer Verantwortung
	Qualifikation [16, 33, 38, 60]	*Keine Vorbereitung auf Pandemiefall *in Ausbildung, Studium, Weiterbildung
		Durchführung neuer Prozeduren, z. T. *ohne ausreichende Einarbeitung*
Soziale Beziehungen	Kommunikation zwischen Pflegenden und Patienten, Bewohnern und Angehörigen [14, 15, 21, 26, 27, 29, 30, 34, 35, 38, 57, 60]	Eingeschränkte Kommunikation und Beziehungsaufbau durch persönliche Schutzausrüstung
		Angespannte Beziehungen
		Gestiegene Erwartungen an Pflegende (v. a. ambulante Pflege)
		Konflikte, Schuldzuweisungen von Angehörigen bis zu verbaler und körperlicher Gewalt durch Umsetzung von Kontaktverboten, häufig wechselnde Regelungen
	Kommunikation im Team und mit Führungskräften [14, 15, 26, 61]	Misstrauen aufgrund von Infektionsangst zu Pandemiebeginn
		Teamkonflikte und nachlassender Teamzusammenhalt
		Unzureichende Kommunikation mit Führungskräften zu Sorgen und Ängsten

Für einzelne Belastungsfaktoren sind Trendaussagen im Pandemieverlauf möglich. Die Mehrzahl der Querschnittstudien berichtet eine Steigerung des Workloads seit Pandemiebeginn bzw. der Anteil der Pflegekräfte, die einer Steigerung zustimmen, wird im Pandemieverlauf größer (u. a. [15, 16, 31, 33, 56, 57]). Die Werte für Arbeitsüberlastung lagen im August bis Oktober und November/Dezember 2020 in Kliniken und stationären Langzeitpflegeeinrichtungen über den Referenzwerten von Vergleichsstichproben vor der Pandemie. Zeitgleich wurden in der stationären Langzeitpflege und in COVID-19-Bereichen der Kliniken mehr Konflikte zwischen Arbeit und Privatleben als in Vergleichsstichproben vor der Pandemie berichtet [35, 41]. Rückblickend gaben Pflegende die niedrigsten Werte für Arbeitsbelastung im Frühjahr 2020 und die höchsten Werte im Dezember 2020 an [20]. In der stationären Langzeitpflege lag die Arbeitsüberlastung im Herbst 2021 (Welle 5) unterhalb der Daten von Vergleichsstichproben aus 2019 [28]. Tendenziell weist das Ergebnis auf eine Entspannung der Situation im Pandemieverlauf hin. Die Daten beruhen aber auf einer sehr kleinen Stichprobe (*n* = 55). Die Belastungswahrnehmung war in der ambulanten Pflege geringer als in Kliniken und stationärer Langzeitpflege, in COVID-19-Bereichen im Vergleich zu Non-COVID-Bereichen höher. Pflegende nahmen eine stärkere Zunahme der Arbeitsbelastung und weniger Erholungszeit als Mediziner wahr [41, 45, 49].

Der Mangel an persönlicher Schutzausrüstung (PSA) und Desinfektionsmitteln einschließlich der Arbeitsintensivierung durch Beschaffungsaufwand wurde in der ersten Pandemiephase und retrospektiv bis April 2020 beschrieben [14, 16, 21, 23, 26, 33, 34, 38, 57]. Im Vergleich war der erlebte Mangel an PSA in Kliniken geringer als in ambulanter Pflege und/oder stationärer Langzeitpflege [16, 33, 56, 57]. Zugleich war das (lange) Tragen der PSA physisch belastend und die Umsetzung der Auflagen bei kognitiv eingeschränkten Bewohnern schwierig [14, 15, 26, 27, 29, 32, 35, 38].

Pandemieassoziierte Sorgen/Ängste, Agieren im Kontext von Tod/Leid der Patienten, Stigmatisierung und moralische Belastung wurden als emotionale Anforderungen betrachtet. Im Frühjahr 2021 lagen die Werte für emotionale Anforderungen bei Pflegenden aus der stationären Langzeitversorgung über den Werten einer Vergleichsstichprobe vor der Pandemie. Ergebnisse zu pandemieassoziierten Ängsten stammen mehrheitlich aus Welle 1 bzw. dem ersten Pandemiejahr [12, 14, 15, 17, 21, 29, 32, 34–36, 45, 48, 54, 57, 59, 61]. Mehr Pflegende waren um die Gesundheit der Angehörigen (> 75 %) und Patienten (bis zu 79 %) als um die eigene Gesundheit besorgt (38–39 %). Pandemiebezogene Sorgen/Ängste waren bei Pflegenden verbreiteter als bei Medizinern. Zukunftssorgen gab es in COVID-19-Versorgungsbereichen häufiger als in den Non-COVID-Bereichen [45–47, 56].

### Beanspruchungsfolgen

Stresserleben, Angst, Depressivität, Burnout/Erschöpfung, (geringes) Arbeitsengagement, Wechselabsichten, Somatisierungssymptome sowie Präsentismus wurden als Beanspruchungsfolgen untersucht. Die Werte der Pflegenden lagen meist oberhalb der Werte von Medizinern, bei Angst und Depressivität Anfang 2020 fast doppelt so hoch. In einer Messreihe mit 4 Messzeitpunkten im Frühjahr 2020 waren Kortisolkonzentrationen aus Haarproben Pflegender höher als die von Medizinern, subjektive Stresslevel unterschieden sich nicht [40]. Personal der COVID-19-Bereiche zeigte sich im Vergleich zu Non-COVID-Bereichen stärker beansprucht [12, 15, 17, 20–23, 26, 31, 35, 36, 38, 40–42, 44, 45, 47–51, 53, 55–61].

Rund die Hälfte der Pflegekräfte nahm eine Steigerung des Stresslevels seit Pandemiebeginn wahr und fühlte sich im Arbeitsalltag gestresst. Ergebnisse mehrerer Querschnittstichproben gleicher Einrichtungen deuten tendenziell auf einen Anstieg von Burnout‑, Angst- und Depressivitätswerten im ersten Pandemiejahr hin. Werte für moralischen Stress (erstes Pandemiejahr), Präsentismus, Wechsel‑/Kündigungsabsichten (August–Oktober 2020, Pflegeheim) und Burnout (Welle 2) lagen oberhalb, Engagementwerte (erstes Pandemiejahr, Klinik) unterhalb der Werte von Vergleichsstichproben vor der Pandemie. 12,7 % der Leitungskräfte gaben an, häufiger krank zur Arbeit zu gehen. In der ambulanten Pflege wurde zu Pandemiebeginn ein höheres Maß an Arbeitsengagement berichtet.

Hohe Stresslevel beschrieben rund 40 % der Pflegenden im ersten Pandemiejahr. Im Frühjahr 2021 berichteten 60 % hohe Werte für moralischen Stress. Auffällige Angstmaße zeigten zwischen 11,0 % und 36,5 %, auffällige Depressionsmaße zwischen 13,8 % und 41,6 %. In gemischten Stichproben sowie in Stichproben aus der stationären Langzeitpflege hatten mehr Pflegekräfte hohe Angst- und Depressionswerte als in Kliniken. Ebenso lagen die Anteile auffälliger Werte für Angst und Depressivität in Querschnittstudien aus späteren Phasen der Pandemie tendenziell über denen früherer Wellen. 21,7 % der Teilnehmenden einer Studie beschreiben im Frühjahr 2021 Suizidgedanken innerhalb der letzten 4 Wochen, 25,3 % eine psychische Störung in der Vergangenheit, 19,6 % eine aktuelle psychische Störung. Ein mittleres bis hohes Burnout-Risiko wurde in den Studien bei 40–56 % der Befragten gefunden. Der Anteil der Pflegekräfte mit häufigen Gedanken an einen Berufswechsel lag Ende 2020 bei 20,3 %, monatlich oder öfter dachten 38,0 % der Pflegenden in gemischten Stichproben und 18,9 % der Pflegenden in Klinikstichproben an eine Kündigung/Berufswechsel. Der Anteil der Personen mit Kündigungs‑/Wechselabsichten war außer in einer Studie [23] in jüngeren Altersgruppen höher. Zum Ende der Pandemie (Winter 2022/2023) gaben 32,7 % der Pflegenden an, bei verbesserten Arbeitsbedingungen im Beruf verbleiben zu wollen. 10,5 % der Pflegefachpersonen hatten die Absicht, in den nächsten 12 Monaten den Beruf zu verlassen. Bleibemotivation war gebunden an die Zufriedenheit mit Karriereoptionen. Die Tätigkeit in der Altenpflege war mit einer um 29 % verringerten Chance auf eine Bleibemotivation assoziiert. Die Schlafqualität war mehrheitlich nicht auffällig beeinträchtigt, 47 % nahmen jedoch eine geringere Schlafqualität seit Pandemiebeginn wahr. Rund 38 % beschrieben eine schlechte Schlafqualität. Positive Beanspruchungsfolgen, wie Erfolgserlebnisse, Zuwachs an Fachwissen und Krisenkompetenz, verbesserte interprofessionelle Zusammenarbeit, wurden zudem berichtet.

### Zusammenhang zwischen Arbeitsbelastung, Ressourcen, Beanspruchungsfolgen

Zusammenhänge zwischen Arbeitsbelastung und Beanspruchung wurden nur in Querschnittstudien untersucht. Die Ergebnisse geben erste Hinweise auf relevante Belastungsfaktoren.

Pandemiebezogene Ängste waren mit mehr Stress, Burnout, Angst und Depressivität sowie einem höheren Risiko für mehr als 10 Krankentage assoziiert. Zusammenhänge mit höheren Stressleveln zeigten sich nur bei Klinikpersonal, jedoch nicht in der ambulanten Pflege. Das Engagement war im Frühjahr 2020 bei Sorge um die eigene Gesundheit reduziert, bei Sorge um die Übertragung auf bzw. um die Gesundheit der Patienten jedoch erhöht [12, 31, 36, 41, 54, 59].

Der Kontakt mit COVID-19-Patienten war mit mehr moralischem Stresserleben, weniger Engagement und höheren Burnout-Werten verbunden [41, 49, 57, 61]. Workload hing mit mehr Angst, einer höheren Chance für auffällige Depressionswerte und moralischem Stress zusammen [31, 57]. Je mehr moralischer Stress berichtet wurde, desto höher waren Angst, Depressivität und Burnout [57]. Höhere allgemeine Stresslevel in der Pandemie waren mit weniger Engagement in der Arbeit verbunden [59]. Wechsel‑/Kündigungsabsichten berichteten v. a. Pflegekräfte in Teilzeit, die sich stärker durch pandemiebezogene sowie allgemeine Belastungsfaktoren beansprucht fühlten und höhere Burnout- sowie Depressivitätswerte hatten [23, 47, 54]. Zugleich waren Depressivität und das Gefühl, eine Last für andere zu sein, mit Suizidgedanken verbunden [20]. Pflegekräfte mit einer hohen Arbeitsfähigkeit dachten seltener an einen Berufs‑/Arbeitgeberwechsel [19]. In qualitativen Erhebungen war Stresserleben u. a. mit Doppelbelastung, Konflikten mit Patienten/Angehörigen, Mehrarbeit und höherer Verantwortung verbunden [14, 34]. Angst wurde als Folge von Stigmatisierung beschrieben und auch mit dem Pandemiebeginn assoziiert, der als Ausnahmezustand wahrgenommen wurde [14, 18, 26, 38, 55].

Als arbeitsbezogene Ressourcen wurden soziale Unterstützung durch Kollegen, Vorgesetzte, Arbeitgeber, Familie, Wertschätzung und Belohnung sowie Autonomie untersucht. Daneben wurden Aspekte der Arbeitsorganisation als Ressourcen beschrieben, z. B. die Absicherung der Kinderbetreuung. Die Betreuung von COVID-19-Erkrankten ging mit ungünstigeren Ressourcenbewertungen einher (u. a. [14, 41]).

Eine bedeutsame Ressource in der Pandemie war soziale Unterstützung, u. a. (digitaler) Austausch von Informationen und emotionale Unterstützung bei Angst/Unsicherheit. Der Zusammenhalt im Team und soziale Unterstützung am Arbeitsplatz hingen zusammen mit mehr Arbeitsengagement, weniger Angst, weniger Depressivität [14, 15, 31, 37]. Ausgehend von Isolationsmaßnahmen deutete sich eine Behinderung sozialer Unterstützungsprozesse an [55]. Auch wurde gegenseitiges Vertrauen, z. B. in die korrekte Einhaltung der Hygienevorschriften, als Variante sozialer Ressourcen beschrieben [14, 39]. Zumeist wurde die Zunahme des Zusammenhalts berichtet [14, 15, 29, 32, 34, 35, 55, 60]. Ein Vergleich der sozialen Beziehungen mit Referenzwerten vor der Pandemie zeigte höhere Ausprägungen in der Pandemie [41].

Pflegefachkräfte, die Zugang zu pandemiebezogenen Schulungsangeboten hatten, fühlten sich eher durch ihre Organisation unterstützt. Organisationale Unterstützung war mit weniger Burnout verbunden [47]. Insgesamt hatten der Zugang zu PSA sowie Informationen und klare Anforderungen einen positiven Effekt auf das Engagement [37]. In gut informierten Gruppen lag der Anteil der Befragten mit auffälligen Angstmaßen 6 Prozentpunkte unterhalb der Gruppe mit niedrigem Informationsgrad [56]. In COVID-19-Versorgungsbereichen waren mittlere Werte für faires Management, Unterstützung durch Vorgesetzte, transformationale Führung höher als in Non-COVID-Bereichen. Transformationale Führung hatte aber keinen Einfluss auf das Arbeitsengagement [37, 41].

Eine hohe Belohnungswahrnehmung war mit weniger Kündigungsabsichten und Krankentagen sowie mehr Engagement assoziiert [37, 54]. Bei Verausgabungs-Belohnungs-Ungleichgewichten war die Chance für Gedanken an einen Berufs‑/Arbeitgeberwechsel bzw. die Kündigungsabsicht 4‑mal höher als in Referenzgruppen [19, 54]. Erlebte Wertschätzung durch Patienten hing zusammen mit mehr Aufgehen in der eigenen Tätigkeit (Flow), Arbeitszufriedenheit sowie weniger Erschöpfung. Jedoch wurde die Wertschätzung durch Patienten 2020 im Vergleich zur Zeit vor der Pandemie als geringer eingeschätzt. Höhere Ausprägungen für Wertschätzung durch die Gesellschaft begünstigten höhere Flowwerte [17]. Ab Frühjahr 2021 wurde retrospektiv eine Abnahme der gesellschaftlichen Wertschätzung nach der ersten Welle der Pandemie wahrgenommen [15, 29, 32]. Im Vergleich zu Medizinern hatten Pflegende eine 2‑mal höhere Chance für Verausgabungs-Belohnungs-Ungleichgewichte, welche 62,8 % der Pflegekräfte betrafen, insbesondere aus Kliniken [19, 39, 41]. Personalmangel und als unzureichend wahrgenommene Erholung waren in den Verausgabungs-Belohnungs-Ungleichgewichten mit Personalmangel und unzureichender Erholung verbunden [39]. Die Freitextangaben einer Studie deuten auch zum Ende der Pandemie auf bestehende Verausgabungs-Belohnungs-Ungleichgewichte hin, da Bereitschaftszeiten und Sonderschichten nicht als ausreichend entlohnt erlebt wurden [24].

Humor, Resilienz und das arbeitsbezogene Kohärenzgefühl (Sense of Coherence – SOC) wurden als personbezogene Ressourcen untersucht. Je höher Humor ausgeprägt war, desto geringer war die Erschöpfung und desto höher das Flowerleben, die Arbeitsleistung und die Arbeitszufriedenheit. Humor hing nicht mit Stresserleben zusammen [17]. Eine hohe Resilienz hing zusammen mit weniger Hoffnungslosigkeit, Stress, Angst, Depressivität, Burnout, Unruhe usw. [20, 52]. Pflegefachkräfte mit einem hohen SOC waren weniger belastet, zeigten weniger Depressions‑/Angstsymptome und waren weniger ausgebrannt. COVID-bezogene Schulungsangebote, höheres Alter, die Wahrnehmung, durch Mehrarbeit einen Beitrag zur Pandemiebekämpfung zu leisten und Patienten fachgerecht versorgen zu können, waren geringfügig mit einem höheren arbeitsbezogenen SOC verbunden [47, 51]. Weitere Ressourcen wurden in den Studien benannt, u. a. Autonomie, Selbstfürsorgekompetenz (Self-care), abwechslungsreiche Arbeitsinhalte, Freizeit [u. a. 13, 38, 41, 50, 55, 60, 61].

## Diskussion

Im Folgenden werden die Ergebnisse zusammenfassend dargestellt und diskutiert. Ausgewertet wurden 50 Studien zu psychosozialer Arbeitsbelastung, Ressourcen und Beanspruchungsfolgen von Pflegepersonal in der COVID-19-Pandemie in Deutschland. Belastbare Aussagen zu kausalen Zusammenhängen oder Verläufen sind aufgrund der Studiendesigns überwiegend nicht möglich. Mehrheitlich wurden Klinikpersonal und der Zeitraum des ersten Pandemiejahres mittels Online-Befragungen betrachtet. Für spätere Zeiträume wurden bisher weniger Studien publiziert, was möglichweise auf Rekrutierungsprobleme oder noch ausstehende Publikationen zurückzuführen ist. Aufgrund der Teilnahme weniger beanspruchter (Healthy Worker Bias) bzw. technikaffiner Personen können systematische Verzerrungen der Ergebnisse nicht ausgeschlossen werden. Jedoch waren Online-Befragungen eine Möglichkeit, überhaupt Daten zu erheben. In der Mehrzahl der Studien wurden potenzielle Störgrößen, z. B. Geschlecht, Alter, Berufserfahrung, vorbestehende psychische Diagnosen und Doppelbelastung, nicht in die Analyse eingeschlossen. Der Begriff Belastung wurde teils für Belastungsfaktoren, teils für Beanspruchungsmaße (z. B. Stresserleben) verwendet, was die Interpretation erschwerte.

### Arbeitsbelastung insgesamt, Beanspruchungsfolgen und Maßnahmenempfehlungen

Vergleichbar den Ergebnissen internationaler Übersichtsarbeiten sowie professions- und settinggemischter qualitativer Längsschnittstudien [63, 64] berichteten Teilnehmende in den gesichteten Studien retrospektiv zumeist eine *Arbeitsintensivierung* seit Pandemiebeginn in Verbindung mit Personalausfällen, der Umsetzung von Schutz‑/Hygienemaßnahmen, der Änderung von Abläufen, höherem Dokumentations- und Kommunikationsaufwand etc. Die niedrigste quantitative Arbeitsbelastung wurde retrospektiv in der Phase des ersten Lockdowns (März 2020) wahrgenommen, in der bspw. Operationszahlen deutlich sanken. Die höchste Arbeitsbelastung, die in Kliniken und Pflegeheimen auch oberhalb präpandemischer Vergleichswerte lag, wurde retrospektiv in der Phase des dritten Lockdowns (12/2020) wahrgenommen. In dieser Phase wurden mehr COVID-19-Ausbrüche bei Pflegeheimbewohnern beobachtet [65–67]. Für die weitere Entwicklung der Arbeitslast ab 2021 finden sich in den qualitativen Daten vereinzelt Hinweise auf eine Entspannung der Situation (in Pflegeheimen) aufgrund von Impfmöglichkeiten und der Lockerung der Kontaktbeschränkungen.

Angaben zur Belegung und qualitative Ergebnisse deuten auf *differierende Belastungsintensitäten in den Organisationsbereichen, Pandemiephasen und Professionen* hin. Im Vergleich zu Non-COVID-Bereichen wurde in COVID-19-Versorgungsbereichen mehr Arbeitsüberlastung berichtet. Pflegende berichteten im Vergleich zu Medizinern mehr Workload, weniger Erholung und ein höheres Risiko für Verausgabungs-Belohnungs-Ungleichgewichte. Arbeitsüberlastung wurde v. a. von Pflegenden beschrieben, die auch häufiger auffällige Werte für Angst und Depressivität beschrieben. Zugleich war Arbeitsüberlastung mit moralischer Beanspruchung assoziiert, da moralische und fachliche Standards z. T. nicht eingehalten werden konnten. In qualitativen Beschreibungen wird auf die Rationalisierung und Priorisierung von Pflegeleistungen, die Depriorisierung von nichtinfizierten Patienten/Bewohnern in der Pandemie hingewiesen. Weitere moralische Konflikte und Dilemmata (z. B. Anspruch an Pflege am Lebensende vs. Rahmenbedingungen der Versorgung) ähnelten denen in der Zeit vor der Pandemie [68].

*Moralischer Stress*, der bei Kontakt mit COVID-19-Patienten höher war, war zudem mit mehr Angst, Depressivität und Burnout verbunden. In einigen Studien wurde die allgemeine Beanspruchung, d. h. Stresserleben in der Pandemie, erfasst, ohne explizit eine Verbindung zur Arbeitsbelastung herzustellen. Pflegende, die insgesamt mehr Pandemiestress erlebten, zeigten sich zugleich weniger engagiert, waren stärker ausgebrannt, depressiv und dachten häufiger an einen Berufs‑/Arbeitgeberwechsel. Das allgemeine Stresslevel in der Pandemie wurde retrospektiv höher als vor der Pandemie erlebt. Längsschnittstudien, die aufgrund der fehlenden professionsspezifischen Aufbereitung nicht in die Übersichtsarbeit einbezogen wurden, fanden jedoch keine wesentlichen Veränderungen der Stresslevel im Verlauf des ersten Pandemiejahres [69].

Ausgehend von den Ergebnissen bleiben *präpandemische Maßnahmenempfehlungen* zur Reduktion des Personalmangels, der (digital gestützten) Reduktion des Dokumentationsaufwandes sowie des moralischen Stresses bedeutsam (u. a. [70, 71]). Wechsel‑/Kündigungsabsichten stellten sich in Abhängigkeit von den Pandemiephasen, Versorgungssettings und eingesetzten Items (Arbeitgeber‑/Berufswechselabsichten, Kündigungsabsichten insgesamt/monatlich) sehr heterogen dar. Ob und wie viele Pflegende den Beruf in bzw. aufgrund der Pandemie tatsächlich verlassen haben, bleibt noch zu eruieren. Verausgabungs-Belohnungs-Ungleichgewichte werden den Ergebnissen folgend durch COVID-Bonuszahlungen nicht ausreichend adressiert und sollten z. B. durch kompetenz-, ausbildungs-, verantwortungsbasierte Vergütungssysteme, Karriereentwicklungsperspektiven und die Stärkung der pflegerischen Autonomie gelöst werden. Dies kann auch zur Steigerung des Arbeitsengagements, der Bleibemotivation und der Arbeitszufriedenheit beitragen (u. a. [19, 24, 37]).

### Pandemiespezifische Belastung, Beanspruchungsfolgen und Maßnahmenempfehlungen

Ferner ergaben sich *pandemiespezifische Belastungsfaktoren*, z. B. der Mangel an PSA, pandemiebezogene Sorgen/Ängste, Kontakt zu COVID-19-Patienten, schnell wechselnde Regelungen, die an Patienten und Angehörige kommuniziert werden mussten. Teilweise sind diese Belastungsfaktoren aus früheren internationalen Untersuchungen bekannt (u. a. [72]). Sowohl der Mangel an PSA als auch die Umsetzung der Hygiene- und Kontaktauflagen begründeten moralischen Stress, da diese u. a. mit emotionalem Leiden von Patienten/Bewohnern, Widersprüchen zwischen Helferrolle und Sorge um die eigene Infektion und Übertragung des Virus auf die Familie oder Patienten einhergingen. In internationalen Übersichtsarbeiten werden vergleichbare Ergebnisse beschrieben [71, 73]. Zudem waren die Befragten zumeist *nicht auf das Agieren im Pandemiefall vorbereitet*, mussten neue, ungewohnte Prozeduren z. T. ohne ausreichende Einarbeitung durchführen, was als stressrelevant beschrieben wurde. Fühlten sich Pflegende dagegen ausreichend geschützt, informiert und hatten Zugang zu pandemiespezifischen Schulungen, waren sie weniger beansprucht. In Vorbereitung auf zukünftige Pandemien kann die Bevorratung von PSA hilfreich sein. Darüber hinaus erscheinen die Entwicklung und Erprobung von Kurzeinarbeitungskonzepten im Falle von Versetzungen, Wiederbeschäftigung und auch für unterschiedliche Krisenszenarien sinnvoll (u. a. [14, 29, 31, 46, 47]). Denkbar wäre die Bildung eines Pools von speziell geschultem Personal, das in regelmäßigen Abständen gemeinsam trainiert wird und im Krisenfall in Spezialbereiche (von Kliniken) versetzt wird. Die Zeit der Einarbeitung könnte so verkürzt, Hilflosigkeitsgefühle könnten reduziert werden. Regelmäßige Schulungen des gesamten Personals könnten die Vorbereitung auf zukünftige Pandemien unterstützen [74]. Einige der in den Studien berichteten Belastungsfaktoren (u. a. Begleitung schwerer Verläufe ohne helfen zu können) erfüllten die Kriterien für traumatische Erfahrungen und können schwerwiegende psychische Folgen nach sich ziehen [75–78]. Vor diesem Hintergrund sollten Einarbeitungskonzepte stärker als bisher auch den Umgang mit diesen – auch im außerpandemischen Arbeitsalltag auftretenden – psychischen Belastungsfaktoren berücksichtigen. Handlungsempfehlungen liegen vor [76, 77, 79]. Sie setzen auf Psychoedukation und soziale Unterstützung, zumeist durch peergestützte Systeme, die im Vorfeld von Krisen zu etablieren sind.

Zudem können Maßnahmen an der *Stärkung sozialer Unterstützung und des Teamzusammenhalts* ansetzen, da diese mit weniger Stress, Depressivität, Angst, mehr Engagement verbunden waren. Im Vergleich zu präpandemischen Referenzwerten nahm Teamzusammenhalt zu, die interprofessionelle Zusammenarbeit verbesserte sich, wenngleich auch Teamkonflikte/-veränderungen beschrieben wurden. Anzunehmen ist, dass die Einführung digitaler Kommunikationstools den gegenseitigen Austausch in Krisensituationen aufrechterhalten und auch im regulären Alltag unterstützend sein kann. Welche Interventionen zur Stärkung des Teamzusammenhalts geeignet und im Pflegeberuf umsetzbar sind, kann zukünftig stärker in den Forschungsfokus rücken. Berücksichtigt werden sollte, dass Versetzungen in den Studien Wechselabsichten begünstigten, was möglicherweise durch den Verlust an Anbindung und Unterstützung im vertrauten Team beeinflusst wurde. Führungskräfte gaben als eine Bewältigungsstrategie die Förderung des sozialen Miteinanders sowie Elemente gesundheitsförderlicher Führung (Transparenz, Wertschätzung etc.) an. Deutlich wurde, dass Führungskräfte selbst hoch belastet waren. Die Sensibilisierung von Führungskräften für die Gestaltung sozialer Austauschprozesse sowie für Möglichkeiten der Selbstfürsorge könnte ein weiterer Baustein der Ressourcenstärkung sein. Ausgehend von einem transformationalen Führungsansatz wurden in den Studien keine Effekte auf Gesundheitsindikatoren beschrieben. Krisenbezogene Führungsmodelle (z. B. Stabsarbeit) betreffen in erster Linie die geordnete Versorgungssteuerung [74]. Welche Führungsaspekte in der Pandemie eine gesundheitsfördernde Wirkung entfalten und wie diese in Führungskräfteschulungen vermittelt werden können, wurde bisher weniger untersucht. Anzunehmen ist, dass die Kommunikation von Risikoinformationen zur Pandemielage durch Führungskräfte einen Einfluss auf die subjektive Gesundheit der Beschäftigten hat. Auf diese Aufgabe, das Management von Krisen insgesamt und die eigene Selbstsorge, sind Führungskräfte vorzubereiten.

Von den Beschäftigten wurde Bedarf an *Resilienz- und Achtsamkeitskursen* in der Pandemie geäußert. Resilienz und Humor waren in den gesichteten Studien mit weniger Burnout, Angst, Depressivität verbunden. Vergleichbare Ergebnisse finden sich in Längsschnitterhebungen für Gesundheitspersonal gemischter Professionen [80]. Ergebnisse systematischer Reviews zu Resilienztrainings bei Gesundheitspersonal zeigen kleine bis moderate Effekte für die Reduktion von Depressivität, Stress und die Steigerung von Resilienz [81]. Die Umsetzung der Schulungsinhalte erfordert zumeist Übung und Wiederholung im Alltag. Maßnahmen sollten an die Gegebenheiten vor Ort (z. B. Erreichung von Mitarbeitenden im Schichtdienst) angepasst sein und effektiv kommuniziert werden. Ein sicheres und positives Lernumfeld und die Überzeugung der Mitarbeitenden, dass die Intervention wirksam ist, können Teilnahme und Umsetzung beeinflussen [82].

Die Maßnahmen erscheinen auch vor dem Hintergrund bedeutsam, dass sich in den gesichteten Studien bei bis zur Hälfte der Befragten auffällige Werte für psychische Beeinträchtigungen zeigten. *Frauen hatten ein 4‑fach höheres Risiko für auffällige Depressivitätswerte* als Männer, Pflegende waren stärker beansprucht als Mediziner. Im Vergleich mit präpandemischen Daten wurde z. B. ein höheres Maß an moralischem Stress und Burnout gefunden. Mehr Pflegende zeigten in späteren Pandemiephasen auffällige Angst- und Depressionswerte. Jedoch kann auf der Basis der Studiendesigns nicht belastbar auf eine Zunahme gesundheitlicher Beeinträchtigungen geschlossen werden. Auch muss berücksichtigt werden, dass in einzelnen Studien bis zu 1/4 eine psychische Störung in der Vergangenheit berichtete und die Prävalenz für irgendeine psychische Störung nach Selbstauskunft bei 20 % lag. Besorgniserregend waren Daten, wonach 21,7 % der Pflegekräfte im Frühjahr 2021 Suizidgedanken innerhalb der letzten 4 Wochen beschrieben. In einer internationalen Längsschnittstudie (2020 bis 2021) wurden hohe Angst- und Depressivitätswerte v. a. bei vorbestehender psychischer Störung gefunden [83]. Grasmann et al. [84] publizierten nach Abschluss dieses Reviews Ergebnisse einer Längsschnittstudie (4 Messzeitpunkte bis 2022) bei Pflegekräften in Deutschland. Angst und Depressivität lagen zu jedem Messzeitpunkt oberhalb der Werte der Allgemeinbevölkerung in der Pandemie. Zudem war im Vergleich mit dem ersten Messzeitpunkt zu jedem weiteren Messzeitpunkt eine signifikant höhere Ausprägung depressiver und Angstsymptome zu konstatieren.

Zur Beurteilung der Entwicklung von Belastung und Beanspruchung und zur Erarbeitung geeigneter Interventionen ist die Förderung langfristig angelegter Beobachtungsstudien sowie von Interventionsstudien unter Verwendung von validierten Instrumenten und Berücksichtigung von Confoundern (z. B. Doppelbelastung) zu empfehlen. Ebenso kann ein betriebsinternes Monitoring i. S. der psychischen Gefährdungsanalyse dabei helfen, *Überlastungsphasen zu erkennen* und gegenzusteuern. Dadurch können auch Bereiche identifiziert werden, die bei vergleichbarer Belastungskonstellation günstigere Ausprägungen der Gesundheitsindikatoren zeigen und als Beispiel für „gute Praxis“ fungieren können. Für Krisenphasen sollten kurze, ggf. App-gesteuerte Tools, die Aufrechterhaltung eines Monitorings unterstützen. Mitarbeitende und Führungskräfte sollten sensibilisiert werden, psychisch beanspruchte Mitarbeitende zu identifizieren und anzusprechen. Dies sollte in einem Klima passieren, welches die Kommunikation erlebter (extremer) Belastung und die Annahme von Hilfe ohne Stigmatisierung ermöglicht. (Extern vernetzte) Angebote der Beratung und Unterstützung bei psychischer Beanspruchung in und außerhalb der Pandemie wurden in den gesichteten Studien ergänzend empfohlen [u. a. 31, 35, 46, 57, 61].

In wenigen Studien wurden auch positive Beanspruchungsfolgen, wie *Wissenszuwachs und Krisenkompetenz* beschrieben. Die gewonnenen Erkenntnisse sollten unter Beteiligung von Pflegefachkräften reflektiert und für die Entwicklung von Handlungsplänen und Ausbildungscurricula für zukünftige Pandemien genutzt werden. Einrichtungs- und behördenübergreifend können in der „pandemiefreien“ Zeit Verabredungen zur Zusammenarbeit in der Pandemie und in weiteren Krisen getroffen werden.

## Fazit

Ausgehend von den Ergebnissen der gesichteten Studien ist von einer pandemiephasen- und -settingspezifischen Kumulation präpandemisch bestehender (z. B. hoher Workload) und pandemiespezifischer Belastungen (z. B. Umsetzung von Hygienebestimmungen) auszugehen. Ebenso werden Phasen und Bereiche der Entspannung bzw. mit geringerer Arbeitslast beschrieben. Ein Einfluss der Belastungsfaktoren auf Gesundheitsindikatoren der Pflegenden deutet sich an, ist auf der Basis der Studiendesigns aber nicht kausal interpretierbar. Ebenfalls kann der Einfluss möglicher Confounder auf Gesundheitsmaße neben der Arbeitsbelastung nicht geschätzt werden. Tendenziell und unter Berücksichtigung von Längsschnittergebnissen professionsgemischter Stichproben verstärken sich gesundheitliche Beeinträchtigungen im Verlauf der Pandemie. Als Ressourcen, zumeist in ihrem direkten Zusammenhang mit Gesundheitsindikatoren untersucht, erwiesen sich etablierte Konzepte der Stressforschung, u. a. soziale Unterstützung, Teamzusammenhalt, Kohärenzgefühl, Resilienz, im Sinne von Stressbewältigungsfähigkeit. Die daraus abgeleiteten Maßnahmen sollten am Abbau präpandemisch bestehender Belastungsfaktoren und der Stärkung von Ressourcen ansetzen, um eine weitere Verschärfung und Belastungskaskaden in zukünftigen Krisen zu vermeiden. Spezifische Belastungsfaktoren in der Pandemie sollten systematisch reflektiert und organisationsinterne sowie -übergreifende Lösungen in Vorbereitung auf weitere Krisensituationen erarbeitet werden.

## Supplementary Information

Tabelle Z1 Suchterme, Tabelle Z2 Eingeschlossene Studien, Tabelle Z3 Studienbeschreibung und Ergebnisse, Abbildung Z1 PRISMA FlowChart

## Footnotes

1 Die DIN EN ISO 10075‑1 ist Teil eines branchenübergreifenden Standards mit Richtlinien zur ergonomischen Gestaltung der Arbeit in Bezug auf die psychische Belastung und definiert u. a. zentrale Begriffe/Konzepte.

2 Aufgrund der besseren Lesbarkeit wird das generische Maskulinum oder die genderneutrale Schreibweise verwendet. Gemeint sind jedoch immer alle Geschlechter.

3 Siehe Ergebnisbericht. Zum Teil wurde kein „echter“ Längsschnitt generiert, da wiederholt teilnehmende Personen nicht identifiziert werden konnten. Teilauswertungen der VOICE-Studie (multizentrische, webbasierte und prospektive Befragung) wurden als Querschnittdaten berücksichtigt.
