# Supplementary material for: Workload and mental health of nursing staff in Germany during the COVID-19 pandemic—a scoping review
Source: Bundesgesundheitsblatt Gesundheitsforschung Gesundheitsschutz. 2024 Dec 3;68(2):130–40. [Article in German] doi: 10.1007/s00103-024-03984-5 (PMC11775038; doi:10.1007/s00103-024-03984-5)
Supplement: Supplementary file 1 — Tabelle Z1 Suchterme, Tabelle Z2 Eingeschlossene Studien, Tabelle Z3 Studienbeschreibung und Ergebnisse, Abbildung Z1 PRISMA FlowChart [file 103_2024_3984_MOESM1_ESM.pdf]

Onlinematerial zum Beitrag "Arbeitsbelastung und psychische Gesundheit von Pflegekräften in Deutschland während der COVID-19-Pandemie – Ein Scoping-Review, Bundesgesundheitsblatt Heft 2/2025"

Tabelle Z1: Suchterme

Datenbank	Suchterm
PubMed	#1 ((Nurse* OR "nursing staff" OR "nurse practitioner*" OR "medical professional*" OR "medical worker*" OR "medical staff*" OR "medical personnel*" OR "medical employee*" OR "hospital personnel*" OR "hospital staff*" OR "hospital worker*" OR "hospital employee*" OR "Health* worker" OR "health* personnel" OR "health* professional" OR "health* employee*" OR "health* staff" OR "emergency department staff*" OR "icu staff*" OR "intensive care unit staff*") AND (corona* OR covid* OR *ncov OR "severe acute respiratory syndrome*" OR sars*) AND ("stress*" OR "risk factor*" OR "burden*" OR "load" OR "burdensome factor*" OR strain OR "demand*" OR "moral distress" OR "moral conflict") AND (German*)) Filters: from (2014/12/1:3000/12/12[pdat])
	#2 ((Nurse* OR "nursing staff" OR "nurse practitioner*" OR "medical professional*" OR "medical worker*" OR "medical staff*" OR "medical personnel*" OR "medical employee*" OR "hospital personnel*" OR "hospital staff*" OR "hospital worker*" OR "hospital employee*" OR "Health* worker" OR "health* personnel" OR "health* professional" OR "health* employee*" OR "health* staff" OR "emergency department staff*" OR "icu staff*" OR "intensive care unit staff*") AND (corona* OR covid* OR *ncov OR "severe acute respiratory syndrome*" OR sars*) AND ("mental health" OR wellbeing OR "mental Disorder*" OR exhaustion OR addiction OR "substance abuse" OR suicide OR "sleep Disorder" OR commitment OR engagement OR "intention to leave" OR "psychological impact" OR "psychiatric symptom*" OR "depress*" OR "anxiety" OR "posttraumatic stress" OR "post-traumatic stress" OR PTSD OR "burnout" OR "burn-out" OR "compassion fatigue" OR "moral injury") AND (German*)) Filters: from (2014/12/1:3000/12/12[pdat])
	#3 ((Nurse* OR "nursing staff" OR "nurse practitioner*" OR "medical professional*" OR "medical worker*" OR "medical staff*" OR "medical personnel*" OR "medical employee*" OR "hospital personnel*" OR "hospital staff*" OR "hospital worker*" OR "hospital employee*" OR "Health* worker" OR "health* personnel" OR "health* professional" OR "health* employee*" OR "health* staff" OR "emergency department staff*" OR "icu staff*" OR "intensive care unit staff*") AND (corona* OR covid* OR *ncov OR "severe acute respiratory syndrome*" OR sars*) AND ("resource* OR resilience OR coping OR resiliency) AND (German*)) Filters: from (2014/12/1:3000/12/12[pdat])
PsycINFO	#4 (nurse OR Nurses OR Nursing staff OR nurse practitioner) AND (german OR germany OR Germans) AND (covid-19 or coronavirus or 2019-ncov or sars-cov-2 or cov-19)

Abbildung Z1: PRISMA FlowChart

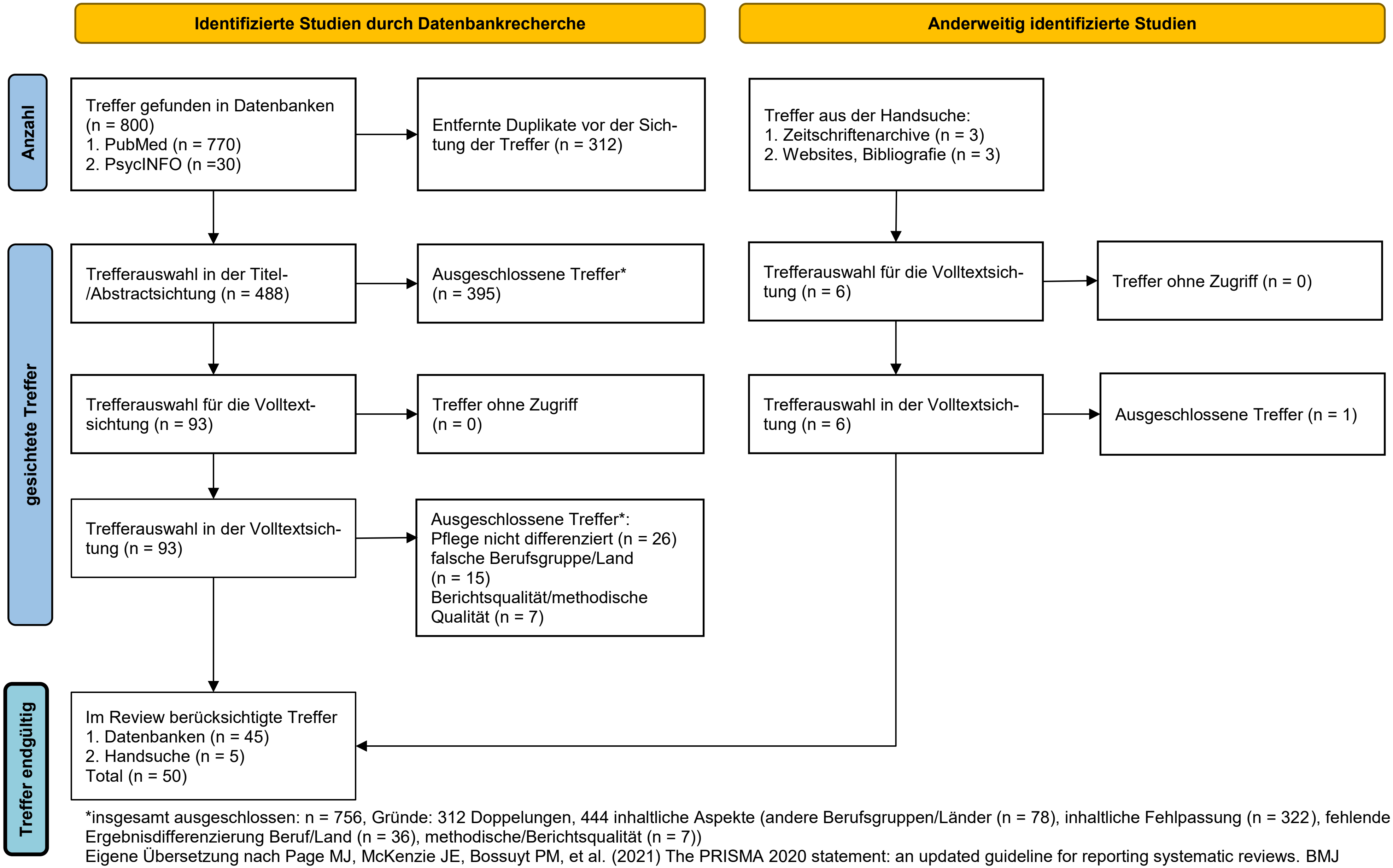

*insgesamt ausgeschlossen: n = 756, Gründe: 312 Doppelungen, 444 inhaltliche Aspekte (andere Berufsgruppen/Länder (n = 78), inhaltliche Fehlpassung (n = 322), fehlende Ergebnisdifferenzierung Beruf/Land (n = 36), methodische/Berichtsqualität (n = 7))
Eigene Übersetzung nach Page MJ, McKenzie JE, Bossuyt PM, et al. (2021) The PRISMA 2020 statement: an updated guideline for reporting systematic reviews. BMJ 372:n71. doi: 10.1136/bmj.n71

Tabelle Z2: Übersicht eingeschlossene Studien (alphabetische Autorenreihung innerhalb der Wellen)

Artikel	Region	Stichprobe				Methode		
		**Größe (n)**	**Anteil Pflegender**	**Setting/ Fachbereich**	**Anteil Frauen %**	**Design**	**Erhebungsmethode**	**Erhebungszeitraum**
**Welle 1 (02.03.2020 – 17.05.2020)**								
[38]	D	397	100%	Klinik, Intensivstation, multizentrisch	100%	Qualitativ, Querschnitt	Qualitativer Websurvey	April 2020
[39]	D	6174	14,9%	Klinik, multizentrisch	77,2% (Gesamtstichprobe)	Quantitativ, Querschnitt	Online-Survey	20.04.-05.07.2020
[40]	München	178	48%	Klinik, unizentrisch	71% (Gesamtstichprobe)	Quantitativ, Längsschnitt	Haaranalysen, Befragung	April-Juli 2020, 4 Messzeitpunkte
[21]	D	525 (RL 12, 1%)	100% Leitungskräfte	Pflegeheim 20%, Hospiz 3,1%, Ambulante Pflege 65%, Betreutes Wohnen 2,9%, Sonstige 4,6%	k. A.	Quantitativ, Querschnitt	Online-Survey	07.04.-25.04.2020
[45]	D	3669 (ca. 92% Ausfüller)	35,9% n = 1317	Klinik, multizentrisch	61% (Gesamtstichprobe=	Quantitativ, Querschnitt	Online-Survey	15.04.-01.05.2020
[48]	D	8071 (RL Pflege 8,9%)	34,7% N Pflege = 1275	Klinik, multizentrisch	75,3% (PK)	Quantitativ, Querschnitt	Online-Survey	20.04.-05.07.2020
[33]	D	824 Pflegeheime (RL 11,2%)	75% Einrichtungsleitung, 25% Pflegedienstleitung	Stationäre Langzeitpflege, multizentrisch	k. A.	Quantitativ, Querschnitt	Online-Survey	28.04. – 12.05. 2020
[51]	D	4324 (RL Pflege ca. 8,1%)	27,1%	Klinik, multizentrisch	76,3% (PK)	Quantitativ, Querschnitt	Online-Survey	20.04.-5.07.2020

Artikel	Region	Stichprobe				Methode		
		Größe (n)	Anteil Pflegender	Setting/ Fachbereich	Anteil Frauen %	Design	Erhebungsmethode	Erhebungszeitraum
[52]	Bonn	1034 (RL Pflege 14,8%)	31,9%	Klinik, unizentrisch	75,5% (PK)	Quantitativ, Querschnitt	Online-Survey	20.04.-05.07.2020
[55]	D	8	60%	Klinik, multizentrisch	k. A.	Qualitativ, Querschnitt-studie	halbstrukturierte leit-fadengestützte tele-fonische Interviews	13.05.-22.05.2020
[56]	D	2224 (RL ?)	67,94%	Klinik, multizentrisch)	86,83% (PK)	Quantitativ, Querschnitt	Online-Survey	10.-31.03. 2020
[53]	D	3293 (RL Pflege: 8%)	34,89%	Klinik, multizentrisch	76,1% (PK)	Quantitativ, Querschnitt	Online-Survey	20.04.-5.07.2020
[58]	D	4724 (RL: ?)	33,34%	Klinik (80,6%, Ambulanz 19,4%), multizentrisch	74,5% (Gesamtstichprobe)	Quantitativ, Querschnitt	Online-Survey	20.04.-05.07.2020
[25]	D	15	100%	k. A.	68,6%	Qualitativ, Querschnitt	Experten/innen Interviews	Frühjahr 2020
[59]	D	1168 (RL ca. 54% Anklicken /Ausfüllen)	80,4%	Klinik (72,9%), stat. Altenpflege (6,4%), amb. Pflege (4,4%), Praxis (4,8), sonstiges (7,8%), multizentrisch	k. A.	Quantitativ, Querschnitt	Online-Survey	13.-27.04.2020
[16]	D	797 (3,5-7,3%)	2/3 Pflegedienstleitung, 1/3 Einrichtungsleitung	Ambulante Pflege (88%), teilstationäre Einrichtungen (12%)	k. A.	Quantitativ, Querschnitt	Teil-strukturierte Online-Befragung	28.04.-12.05.2020

Artikel	Region	Stichprobe				Methode		
		Größe (n)	Anteil Pflegender	Setting/ Fachbereich	Anteil Frauen %	Design	Erhebungsmethode	Erhebungszeitraum
[61]	Augsburg	110 (RL: ?)	68,18%	Klinik, unizentrisch	70% (Gesamtstichprobe)	Quantitativ, Querschnitt	Online-Survey	März-April 2020
**Sommerplateau 2020 (18.05.2020-27.09.2020)**								
[17]	D	174 (58% Abschluss FB, RL?)	100%	Gemischt, k. A.	92,5%	Quantitativ, Querschnitt	Online-Survey	Juni-Juli 2020
[27]	D	n = 78 Interviews in 43 Einrichtungen	25 Einrichtungsleitung, 15 Pflegedienst, 28 Wohnbereichsleitung, 10 Pflegefachpersonen	Stationäre Langzeitpflege, multizentrisch	k. A.	Qualitativ, Querschnitt	Semistrukturierte Telefoninterviews	Juni-Juli 2020
[30]	D	n = 78 Interviews in 43 Einrichtungen	25 Einrichtungsleitung, 15 Pflegedienst, 28 Wohnbereichsleitung, 10 Pflegefachpersonen	Stationäre Langzeitpflege, multizentrisch	k. A.	Qualitativ, Querschnitt	Semistrukturierte Telefoninterviews	Juni-Juli 2020
[14]	Hamburg	15	100%	Ambulante Pflege	80%	Qualitativ, Querschnitt	halbstrukturierte telefonische Interviews	Mai-Juni 2020
[34]	D	78	40% Management Pflegeheim, 38 Stationsleitungen	Stationäre Langzeitpflege, multizentrisch	k. A.	Qualitativ, Querschnitt	Halbstrukturierte telefonische Interviews	Mai-Juli 2020

Artikel	Region	Stichprobe				Methode		
		**Größe (n)**	**Anteil Pflegender**	**Setting/ Fachbereich**	**Anteil Frauen %**	**Design**	**Erhebungsmethode**	**Erhebungszeitraum**
**Welle 2 (28.09.2020 – 28.02.2021)**								
[37]	D	1027 (39% Full Response)	100%	Klinik 76,7%, Altenpflegeeinrichtung 13,3%, Ambulante Pflege 8,1%, Sonstige 1,9%	83,1%	Quantitativ, Querschnitt	Online-Survey	20.11. – 07.12.2020
[26]	D	510	100%	Stationäre Langzeitpflege, multizentrisch	k. A.	Qualitativ, Querschnitt	Web-basierter qualitativer Survey	31.10.2020 – 25.02.2021
[41]	D	1027 (49,4% Teilnahme von allen Aufrufenden)	100%	Klinik 76,7%, Altenpflegeeinrichtung 13,3%, Ambulante Pflege 8,1%, Sonstige 1,9%	83,1%	Quantitativ, Querschnitt	Online-Survey	20.11. – 07.12.2020
[13]	Süddeutschland	115 (57,5%)	100%	Ambulante Pflege	81,7%	Mixed-Methods, Querschnitt	Fragebogen (Paper-Pencil), Audio-Tagebuch	September – Oktober 2020
[31]	D	811	100%	Stationäre Langzeitpflege	90,6%	Quantitativ, Querschnitt	Online-Survey	15.11.2020 – 28.02.2021
[44]	D	484 (RL: ?)	49,8%	Klinik, multizentrisch	80,4% (Gesamtstichprobe)	quantitative Querschnittstudie	Online-Survey	November 2020-März 2021
[46]	Hannover	1163	39,6%	Klinik, unizentrisch	73,2%	Multi-Method, Querschnitt	Online-Survey	November 2020-Februar 2021
[22]	D (GB, IT, SE)	120 D	74% Pflegekräfte, 26% Management	Keine Differenzierung	80,8%	Quantitativ, Querschnitt	Online-Survey	7.10.2020 – 17.12.2020

Artikel	Region	Stichprobe				Methode		
		**Größe (n)**	**Anteil Pflegen-der**	**Setting/ Fachbereich**	**Anteil Frauen %**	**Design**	**Erhe-bungs-methode**	**Erhe-bungs-zeitraum**
[47]	Hessen	872 (RL: ?; Ab-schlus s 73%)	68,23%	Klinik, multizentrisch	M=76,1 % W=23,7 % Zahlen-dreher?	Quantitativ, Querschnitt	Online-Survey	Dezember 2020-Jan-uar 2021
[23]	D	301 (ca. 12,4%)	100%	Pflegeheim, Ambulante Pflege, Hospiz	69%	Quantitativ, Querschnitt	Online-Survey	Dezember 2020 – Januar 2021
[35]	Brandenburg	Befra-gung: 177 (RL 11,5%) , Inter-views: 15	Befra-gung: 58,7% Pflege-kräfte, 41,3% Pflegeas-sisten-ten/innen Inter-views: 26% Ma-nager*in	Stationäre Lang-zeitpflege, mul-tizentrisch	Befra-gung: 82,9%, Inter-views: 80%	Mixed Me-thods (Befra-gung, Inter-views), Querschnitt	Fragebo-gen (Paper-Pencil), se-mistruktu-rierte Tele-fon-/Onlin-einterviews	Befragung: August-Ok-tober 2020; Interviews: Juni 2021
[36]	Brandenburg	195 (RL 11,2%)	Alten-pflege 34,4%, Altenpfle-gehilfe 19,5%, Pflege-hilfe 16,4%, Gesund-heits- und Kranken-pflege 16,4%, Betreu-ung 7,2%, 5,1% Freiwillige u.a. Sons-tige	Stationäre Langzeitpflege	81,5%	Quantitativ, Querschnitt	Frageboge n (Paper-Pencil)	August-Dezember 2020

Artikel	Region	Stichprobe				Methode		
		Größe (n)	Anteil Pflegen-der	Setting/ Fachbereich	Anteil Frauen %	Design	Erhe-bungs-methode	Erhe-bungs-zeitraum
**Welle 3 (01.03.2021-13.06.2021)**								
[29]	Rheinland-Pfalz	10	100% 90% Pflegefac hkraft, 10% Al-ten-pflegehilfe	Stationäre Al-tenpflege, multizentrisch	90%	Qualitativ, Querschnitt	Telefon-interview	März – Juni 2021
[19]	D	2689 (19.08 2 Mails +wei-tere An-spra-che, RL bei ca. 14%)	100%	teil-stationäre Pflege 22,1%, ambulante Pflege 12,4%, Tagespflege 1,1%, Klinik 56%, WG 1,1% Sonstige 7,4%	75,1%	Quantitativ, Querschnitt	Online-Survey	12.03. – 12.05.2021
[20]	D	1311 (RL: ?)	100%	k. A.	96,9%	Quantitativ, Querschnitt	Online-Survey	Februar – April 2021
[32]	Berlin	Inter-views n = 18	100%	Stationäre Altenpflege	Inter-views 72%	Mixed Methods, nur Interviews berücksich-tigt, Quer-schnitt	Leitfaden-interview	Juni 2021
[49]	Augsburg	286	53,15%	Klinik, unizentrisch	64%	Quantitativ, Querschnitt	Befragung	März-April 2021
[54]	D	757 (RL: ?)	100%	Klinik, multizentrisch	76,9%	Quantitativ, Querschnitt	Online-Survey	Mai-Juli 2021
[57]	D	246 RL 7%	50,81%	Klinik, multizentrisch	81,6% (PK)	Quantitativ, Querschnitt	Online-Survey	März-Juli 2021

Artikel	Region	Stichprobe				Methode		
		Größe (n)	Anteil Pflegender	Setting/ Fachbereich	Anteil Frauen %	Design	Erhebungsmethode	Erhebungszeitraum
[60]	Köln	32	66%	Klinik/ Intensivstation, unizentrisch	66% (PK)	Datenerhebung im Rahmen Teamintervention, Querschnitt	Gruppendiskussion	Juli 2021
**Sommerplateau 2021 (14.06.2021-01.08.21)**								
**Welle 4 (02.08.2021-26.12.2021)**								
**Welle 5 (27.12.2021-29.05.2022)**								
[28]	Nordrhein-Westfalen	2019 n = 55, 2022 n = 58 (RL: 59%-62%)	100%	2 Pflegeheime, Schwerpunkt Palliativversorgung	76,4% - 75,9%	Quantitativ, Studie mit zwei Messzeitpunkten (Trendanalyse)	Fragebogen (Paper-Pencil)	t1 3.10.2019-14.11.2019<br>t2 25.04.2022 - 03.06.2022
**Welle 6 (30.05.2022 -)**								
[15]	D	976 (16% voll ausgefüllt)	100%	Ambulante Pflege	82,7%	Quantitativ, Querschnitt	Online-Survey	Juni – Juli 2022
[24]	Bayern	2572 (RL: k.a.)	100%	57,9% Klinik, 23,9% Pflegeheim, 15,1% ambulante Pflege	81,4%	Quantitativ, Querschnitt	Online-Survey	Oktober 2022-Januar 2023
**Gemischt - Wellenübergreifend**								
[12]	D	166 (RL 28,2%)	100%	Ambulante Pflege	65,1%	Quantitativ, Querschnitt	Online-Survey	Mai 2020-Mai 2021 (Welle 1-3)
[42]	Hamburg	1792	20,3%	Klinik	72,4%	Quantitativ, Follow-up/Längsschnitt	Fragebogen (Paper-Pencil)	April und August 2020 (Welle 1 +2)

Artikel	Region	Stichprobe				Methode		
		Größe (n)	Anteil Pflegender	Setting/ Fachbereich	Anteil Frauen %	Design	Erhebungsmethode	Erhebungszeitraum
[43]	D	1231	30%	Klinik (Onkologie, Psychiatrie)	77,8% (PK)	Quantitativ, Querschnitt	Fragebogen (Paper-Pencil)	Oktober 2020-Juni 2021 (Welle 2-3)
[50]	Hessen	26	100%	Klinik	73,1%	Qualitativ, Querschnitt	Telefonische qualitative Experteninterviews	November 2020 – Juli 2021 (Welle 2-3)
**Keine Angaben**								
[18]	D	16	100%	Klinik, Pflegeheim	k. A.	Qualitativ, Querschnitt	Leitfadengestützte teilstandardisierte Interviews	k. A.

Anmerkung: D = Deutschland, GB = Großbritannien, IT = Italien, SE = Schweden

Tabelle Z3: Studienbeschreibung und Ergebnisse

Autoren, Zeitraum, Region	Studienziel, Methode, Stichprobenumfang	Messinstrumente	Hauptergebnisse
**Setting Ambulante Pflege**			
[12], 5/2020-5/2021, Deutsch-land	• Beschreibung Stresserleben, Schlafqualität, pandemieassoziierte Sorgen, Arbeitsengagement<br>• Zusammenhang Stresserleben, Schlafqualität, Arbeitsengagement,<br>• Zusammenhang pandemieassoziierte Sorgen und Stresserleben<br>• Online-Befragung<br>• n = 171	• PSS-10<br>• Schlafqualität seit Pandemiebeginn/ letzte 4 Wochen (analog PSQI)<br>• COPSOQ (Arbeitsengagement)<br>• Pandemiebezogene Ängste und Sorgen, Bedürfnisse, Gesundheitliche Konsequenzen, Behandlung, Bedarf psychologische Unterstützung, Vorbereitung Arbeitgeber	**Belastung**<br>• 65,7% Pandemie-Sorgen, 70,5% Sorge Ansteckung Familie, 65,7% Sorge eigene Ansteckung Arbeitskontext, 62% Sorge Isolation von Familie/Umfeld, 44,6% Sorge Leistungsfähigkeit, 60% unbesorgt über gesundheitliche Folgen einer Infektion, 45,7% keine gute Vorbereitung des Arbeitgebers auf Pandemie, mehrheitlich kein Bedarf an psychologischer Beratung<br>**Beanspruchung**<br>• moderat hohes Stressniveau, 1/3 hohe Werte Stresserleben, 50,6% eher/voll/ganz Zustimmung Zunahme Stresserleben seit Pandemiebeginn<br>• 37,9% schlechte/sehr schlechte Schlafqualität (62% gute/sehr gute Schlafqualität), 47% eher/voll Zustimmung Verschlechterung Schlafqualität seit Pandemiebeginn (52,4% keine Veränderung)<br>• Hohe Ausprägungen Arbeitsengagement<br>**Zusammenhänge**<br>• Stresserleben positiv assoziiert mit geringerer Schlafqualität, geringeres Arbeitsengagement<br>• pandemiebezogene Sorgen/Bedenken kein Prädiktor für Stresserleben
[13], 9/2020-10/202, Süddeutsch land	• Resilienz, Bedeutung der Arbeit<br>• Art/Häufigkeit freudiger Arbeitsmomente<br>• Unterschiede in Bezug auf Geschlecht, Alter, Muttersprache, Region und Beruf<br>• Mixed Methods<br>• qualitativ: N=23, quantitativ: N = 115	• Skala für berufliche Resilienz<br>• Skala zur beruflichen Sinnhaftigkeit<br>• Audiotagebuch: freudige Momente	**Ressourcen:**<br>• höhere Werte für problemfokussierte als für emotionsfokussierte Resilienzskalen<br>• höhere Resilienzmaße für Frauen<br>Freudvolle Momente:<br>• soziale Beziehungen (Wertschätzung durch Patienten, Team, Angehörige; Unterstützung Kollegium, Angehörige; positive Emotionen der Patienten, Teamkultur)<br>• Arbeitsinhalt (abwechslungsreiche spannende Arbeitsinhalte, Bewältigung herausfordernder Anforderungen, leichter Arbeitstag ohne Komplikationen)<br>• Arbeitsorganisation (kein Zeitdruck, früheres/rechtzeitiges Arbeitsende, späterer Arbeitsbeginn)<br>• Arbeitsumfeld (ruhige Verkehrssituation, gutes Wetter, gut vorbereitetes Auto)<br>• Selbstfürsorge (aktive Mittagspause, Selbstermutigung, Gefühl, fit zu sein)
[14], 5/2020-	• pandemieassoziierte Arbeitsbelastungen, arbeitsbezogene		**Belastung**

Autoren, Zeitraum, Region	Studienziel, Methode, Stichprobenumfang	Messinstrumente	Hauptergebnisse
6/2020, Hamburg	Ressourcen, Beanspruchungsfolgen, Bewältigungsstrategien, Unterstützungsbedarf, Gesundheitsverhalten<br>• halbstrukturierte telefonische Interviews<br>• n = 15		• Arbeitsorganisation (u.a. Einspringen, schriftliche Übergabe Risiko für Missverständnisse)<br>• Quantitative Arbeitslast (u.a. höhere Arbeitszeit, neue pandemiespezifische Aufgaben, Wartezeit Ärzte/Apotheken)<br>• Neue Arbeitsaufgaben (Umgang mit infizierten Patienten bei fehlenden Standards, hohe Verantwortung, Selbstständigkeit)<br>• Arbeitsumfeld und Arbeitsmittel (Einhaltung des Mindestabstandes z.T. nicht möglich, Maskenpflicht, Pandemiebeginn Fehlen bzw. Beschaffungsprobleme PSA)<br>• Soziale Beziehungen (Umgang mit Ängsten/Sorgen und aggressiven Verhaltensweisen der Patienten, Angst vor gegenseitiger Ansteckung/Misstrauen unter Kollegen, erschwerte Kommunikation durch Masken, Aufklärung der Angehörigen/Patienten, mangelnde Kommunikation mit Führungskräften über Sorgen und Ängste)<br>• Emotionale Anforderungen (Angst, Einsamkeit, Traurigkeit der Patienten, Sterben/Leid, Emotionsarbeit, Angst vor Ansteckung/Übertragung, Isolation, finanzielle Unsicherheit, Kinderbetreuung, Streitigkeiten im sozialen Umfeld, Diskriminierung durch Patienten, Angehörige, Öffentlichkeit)<br>**Beanspruchung**<br>• Depressive Symptome, Angst/Furcht, Beeinträchtigung der individuellen Leistungsfähigkeit, Müdigkeit, erhöhtes Stressempfinden<br>**Ressourcen**<br>• Soziale Ressourcen: Teamgeist/-spirit, soziale Unterstützung, Vertrauen, Wertschätzung durch Vorgesetzte und/oder Kollegen, Mitmenschen<br>• Allgemeine Arbeitsaspekte: Unterstützung Arbeitgeber, Kinderbetreuung, Einhaltung Hygienestandards, Verfügbarkeit PSA<br>• Kommunikation: Austausch von Informationen, Nutzung digitaler Tools
[15], 6/2022-7/2022, Deutschland	• Erfassung pandemieassoziierter Veränderungen der Arbeitssituation<br>• Unterschiede zwischen unterschiedlichen Trägern der ambulanten Pflege<br>• Online-Befragung<br>• n = 976	• Einzelitem Veränderungen der Arbeitssituation seit Pandemiebeginn<br>• Work-Intensification-Scale der Intensification of job demands scale	**Belastung**<br>• 87% Steigerung Workload (68%-76% weniger Pausen, Arbeitstätigkeiten gleichzeitig verrichten, nicht ausreichend Zeit)<br>*Freie Angaben*<br>• Arbeitsorganisation (krankheits-, quarantänebedingter Personalausfall, impfpflichtbedingte Kündigungen, zusätzlicher Aufwand durch Schutzkleidung, Bürokratie)<br>• Veränderungen der Arbeitsinhalte/-Aufgaben (u.a. unklare, wechselnde, widersprüchliche Vorschriften/Handlungsempfehlungen)

Autoren, Zeitraum, Region	Studienziel, Methode, Stichprobenumfang	Messinstrumente	Hauptergebnisse
			• emotionale Belastungsfaktoren (Infektions-/Übertragungsangst, Einsamkeit/Ängste der Patienten, Konflikte /Aggression/Diskussionen/Fragen der Patienten/Angehörigen, gestiegene Erwartungen Patienten/Angehörige, mangelnde Hygiene-/Schutzcompliance Patienten/Angehörige<br>• schlechtere Kommunikation im Team, erschwerte Kommunikation durch Masken, physische Belastung durch PSA, fehlende Refinanzierung Testungen/zusätzliche Fahrten<br>• Beschäftigte in Pflegediensten privater Trägerschaft erleben weniger COVID-19-bedingte Veränderungen als Beschäftigte in Diensten in freier Trägerschaft<br>**Beanspruchung**<br>• mehr Kompetenz im Krisenmanagement<br>**Ressourcen (freie Antworten)**<br>• Abnahme gesellschaftlicher Wertschätzung
[16], 4/2020-5/2020, Deutschland	• Laborbestätigte COVID-Verdachtsfälle in ambulanten/teilstationären Pflegediensten<br>• Personelle/materielle Ressourcen<br>• Bewältigungshandeln<br>• Unterstützungswünsche<br>• Versorgungssituation<br>• Online-Survey<br>• N = 797	• Literaturbasierte Entwicklung von Items zu Strukturmerkmalen der Einrichtung, COVID-19-Vorkommen, Auswirkungen der Pandemie, Wünsche an die Politik	**Belastung – Ambulante Pflege**<br>• 43,0% Personalausfall bis 10%, 42,7% kein Personalausfall<br>• 63,8% Arbeitsmehraufwand, im Mittel 40min/Schicht<br>• 8,5% Kurzarbeit; 45,6% ohne Veränderungen im Personalmanagement bei ausreichendem Personal; 25,1% Umverteilung Stammpersonal<br>• 65,3% nicht auf pandemische Situationen vorbereitet in Ausbildung/Studium<br>• Mangel an Flächendesinfektion, PSA (34,3% bis 55,6%), 1 Woche vor Befragung noch 24% für PSA, 10,9% für Flächendesinfektionsmittel<br>**Belastung – Teilstationäre Einrichtungen**<br>• 58,3% nicht auf pandemische Situationen vorbereitet<br>• 28,1% Personalausfall, 18,4% kein verändertes Personalmanagement, 32,2% Kurzarbeit<br>**Bewältigung – ambulante Pflege**<br>• 3/4 Schulungen für Mitarbeiter, Patienten/Angehörige, Abwesenheitsregelungen bei COVID-typischen Symptomen, 28,8% Regelungen für Risikogruppen<br>• Gründung von Krisenteams, Personalmanagement, Klinisches Monitoring Patienten, Aufnahmestopp, Aufstockung über Ehemalige, Pflegepool, Pflegereserve-Initiative kaum genutzt<br>**Forderungen Politik**

Autoren, Zeitraum, Region	Studienziel, Methode, Stichprobenumfang	Messinstrumente	Hauptergebnisse
			• hohe (≤ 50%) Zustimmung: COVID-Prämie für Pflegende/Rückkehrer, Einsatz Berufsaussteiger, ausländische Kräfte; regelmäßige Testung Pflegender, Beratungen durch Gesundheitsämter, Reduktion Dokumentationsaufwand, Home-Office-Lösungen für administrativ Tätige<br>• hohe Zustimmung (≤90%): Kostenübernahme Sachmittel, Zurverfügungsstellung PSA, bundeseinheitliche Handlungsempfehlungen, Aussetzung externer Prüfungen
**Gemischte Studiensettings**			
[17], 6/2020-7/2020, Deutschland	• Veränderungen Stresserleben, Flow, Arbeitszufriedenheit, Lebenszufriedenheit, Zufriedenheit mit der Arbeitsleistung, Wohlbefinden prä-/postpandemisch (retrospektive Beurteilung)<br>• Puffereffekt von Humor, Wertschätzung auf Stress, Flow, Arbeitszufriedenheit, Lebenszufriedenheit, Zufriedenheit mit der Arbeitsleistung, Wohlbefinden<br>• Online-Survey<br>• N = 174	• Einzelitems aktuelles Stresserleben; Workload in der Pandemie; Zufriedenheit Arbeitsleistung, Arbeit, Leben, Wohlbefinden; pandemiebedingte Sorgen/Ängste, Wertschätzung Patienten/Gesellschaft<br>• Irritation Scale (Subskala Emotionale Irritation)<br>• MBI -D (Subskala Emotionale Erschöpfung)<br>• Flow Frequency Scale<br>• Sence of humor Scale<br>• Subjektive Einschätzung vor/in der Pandemie: Stresserleben, Irritabilität, Emotionale Erschöpfung, Zufriedenheit, Wohlbefinden, Flow, Wertschätzung	**Belastung**<br>• Sorge um Gesundheit der Familie, Freunde; Werte geringer für Sorge um eigene Gesundheit, wirtschaftliche Zukunft<br>• 66 % Zunahme Workload, 18 % keine Veränderung, 16% Abnahme<br>**Beanspruchung**<br>• Werte für Stresserleben, Irritabilität, Emotionale Erschöpfung im Vergleich zu Angaben für präpandemische Zeit höher<br>• Werte für Zufriedenheit mit Arbeit, Leben, Arbeitsleistung, Wohlbefinden, Flow im Vergleich zu Angaben für präpandemische Zeit geringer<br>**Ressourcen**<br>• Werte für Wertschätzung durch Gesellschaft höher, durch Patienten geringer im Vergleich zu Angaben für präpandemische Zeit<br>• Hohe mittlere Werte für Humor<br>**Zusammenhänge**<br>• Humor insgesamt mit weniger Erschöpfung und mehr Flow assoziiert<br>• Einzelne Humorfacetten (Freude an Humor, Humor im Alltag, Humor unter Stress) mit höheren Zufriedenheitsmaßen für Arbeit, Arbeitsleistung und weniger emotionaler Irritation in Pandemie verbunden<br>• Wertschätzung durch Patienten mit weniger Erschöpfung, mehr Flow, mehr Arbeitszufriedenheit verbunden<br>• Wertschätzung durch Gesellschaft mit mehr Flow verbunden
[18], keine Angabe Zeitraum, Deutschland	• Art und Auswirkungen von pandemiebedingten Stigmatisierungserfahrungen<br>• Präventionsmaßnahmen, Interventionen		**Belastung**<br>• Stigmatisierungsquellen: Freunde/Bekannte, Familienangehörige, Fremde, Vorgesetzte, Kollegen, Patienten, Angehörige, öffentliche Berichterstattung, Selbststigmatisierung<br>• Stigmatisierungsanlässe privater Bereich: Angst vor Ansteckung, Unsicherheit/mangelndes Wissen über Erkrankung/Arbeitstätigkeit

Autoren, Zeitraum, Region	Studienziel, Methode, Stichprobenumfang	Messinstrumente	Hauptergebnisse
	• Leitfadengestützte teilstandardisierte Interviews<br>• N = 16		• Stigmatisierungsanlässe beruflich: krankheitsbedingter Ausfall, Impfskepsis, Schuldzuweisung für COVID-Ausbruch auf Station<br>• Stigmatisierungsformen: Distanzierung, Kontaktvermeidung, Ausgrenzung (auch assoziativ), Vorwürfe, Unterstellung einer Erkrankung, Beobachtung und Kontrolle (Ordnungsamt in Quarantäne); Bloßstellung, Missgunst (wegen Coronabonus), Verhöhnung durch Fremde, Schuldgefühle, Belastung durch eigene Infektiosität<br>**Beanspruchung**<br>• Enttäuschung, Verletzung, Ausgeschlossen-/Allein-Sein, Angst vor Kündigung, Ärger, Wut, Anspannung<br>**Bewältigung**<br>• Verschweigen Berufstätigkeit, Kontaktvermeidung/-Abbruch, bewusste Kontaktpflege, Austausch mit Nahestehenden bzw. Psychotherapeuten, Konfrontation, Aufklärung, Verständnis, Sport, Rauchen, Aufmerksamkeitslenkung, Abgrenzung, Impfen
[19], 3/2021-5/2021, Deutschland	• Arbeitsfähigkeit<br>• Verhältnis Aufwand/Belohnung<br>• Wechselabsichten<br>• Einflussfaktoren auf Arbeitsfähigkeit, Aufwand-/Belohnungsverhältnis, Wechselabsichten<br>Online-Survey<br>N = 2689	• WAI<br>• ERI-Fragebogen<br>• Wechselabsichten	**Belastung & personale Risiken**<br>• ERI-Ratio: 1,7; mehr Aufwand als Belohnung in allen Gruppen, Pflegende aus Kliniken höchste ERI-Ungleichgewichte<br>• Frauen höhere ERI-Ungleichgewichte als Männer<br>• Übermäßiges Pflichtgefühl/Overcommitment im oberen Bereich<br>**Beanspruchung**<br>• im Mittel gute Arbeitsfähigkeit, weibliche Pflegende geringere Arbeitsfähigkeit<br>• höhere Arbeitsfähigkeit bei Klinikpersonal, Arbeitsfähigkeit nimmt mit Berufsjahren ab<br>• Berufswechsel: 38% monatlich/häufiger, Arbeitgeberwechsel: 30,6% monatlich/häufiger<br>**Ressourcen**<br>• Belohnung im mittleren Bereich<br>**Zusammenhänge**<br>• Ungünstiges ERI-Ratio, geringere Arbeitsfähigkeit, jüngeres Alter mit Wechselabsichten Beruf assoziiert<br>• Ungünstigere ERI-Ratio mit Wunsch nach Wechsel Arbeitgeber assoziiert, geringere Arbeitsfähigkeit, kürzere Beschäftigungsdauer mit geringeren Arbeitgeber-Wechselabsichten assoziiert

Autoren, Zeitraum, Region	Studienziel, Methode, Stichprobenumfang	Messinstrumente	Hauptergebnisse
			• weibliche Pflegende geringere Arbeitsfähigkeit als Männer, Menschen diversen Geschlechts und Frauen ungünstigere Aufwands-Belohnungsverhältnisse
[20], 2/2021-4/2021, Deutschland	• Angst, Stress, Depressivität, Burnout, Suizidgedanken<br>• Einfluss von Resilienz und Kontakt zu COVID-Patienten auf psychische Beanspruchungsmaße<br>• Retrospektive Beurteilung der Veränderung von Belastung und Beanspruchungsmaßen über ein Jahr<br>• Online-Survey<br>• N = 1311 (25,3% mit psychischer Störung in Vergangenheit, 19,6% mit aktueller psychischer Störung davon 49,6% aktuell in Behandlung)	• BAT,<br>• DASS-21, DESC, BHS<br>• BAM<br>• INQ<br>• SDES<br>• SSEV<br>• RS-13<br>• Je 1 Einzelitem zur subjektiven Beurteilung der beruflichen Belastung und psychischen Belastung (Beeinträchtigungen zwischen den Wellen)	**Belastung**<br>• 32% mehr Überstunden in Pandemie<br>• retrospektiv beurteilte subjektive berufliche Belastung während der ersten Schließung im März 2020 am niedrigsten und während der dritten Schließung im Dezember 2020 am höchsten<br>**Beanspruchung**<br>• 41,5% auffällig Depressivität, 25,9% hohes Burnout-Risiko, 26,8% mittleres Burnout-Risiko<br>• 21,7% Suizidgedanken letzte 4 Wochen, 44,5% Suizidgedanken Lebenszeit<br>• 12,6 % mindestens ein Suizidversuch Lebenszeit<br>**Ressourcen**<br>• 32,1% hohe Maße Resilienz<br>**Zusammenhänge**<br>• Hohe Resilienz im Vergleich zu geringerer Resilienz assoziiert mit weniger Hoffnungslosigkeit, Stress, Angst, Depressivität, Burnout, Gefühl eine Last für Familie, Freunde oder Gesellschaft zu sein, Erregung/Unruhe, mangelndes Zugehörigkeitsgefühl, Gefühle von Niederlage und Gefangensein<br>• keine Unterschiede für Vergleich von Gruppen mit/ohne Kontakt zu COVID-Patienten für Resilienz oder Suizidalität<br>• Suizidgedanken stehen im Zusammenhang mit höheren Maßen für Depressivität, Suizidversuchen in der Lebenszeit, Erregung/Unruhe, Gefühl eine Last für Familie, Freunde oder Gesellschaft zu sein (47% Varianzaufklärung)
[21], 4/2020, Deutschland	• Belastung in Pflege- und Hospizeinrichtungen aus Sicht der Leitungskräfte<br>• Veränderungen allgemeine Belastung<br>• Gesundheitliche Veränderungen (Präsentismus, allgemeiner Gesundheitszustand)<br>• Online-Survey	• Einzelitemanalyse, literaturbasiert entwickelt	**Belastung**<br>• Ca. 70% stark/sehr stark belastet durch Sorge um Infektion Pflegebedürftiger/Mitarbeitende, Beschaffung/Verbrauch von PSA/Hygienematerial<br>• Ca. 50%-60% stark/sehr stark belastet durch Einnahmeausfälle, Finanzmittelausstattung Pandemiebewältigung<br>• Ca. 50% stark/sehr stark belastet durch widersprüchliche/intransparente Informationen, Einhaltung Kontaktverbot für Angehörige<br>• 42% stark/sehr stark belastet durch Überlastung der Mitarbeitenden

Autoren, Zeitraum, Region	Studienziel, Methode, Stichprobenumfang	Messinstrumente	Hauptergebnisse
	• N = 525		• 1/3 stark/sehr stark belastet durch Arbeitsintensität/ -Verdichtung, hohe Erwartungen der Angehörigen, hausärztliche Versorgung, Dienstbesprechungen<br>• größte Belastung: Sorge vor Infektion, Beschaffung PSA, Hygienevorschriften, Widersprüchlichkeit/Intransparenz von Informationen, Einnahmeausfälle - Belastungskaskade<br>• Zunahme der Belastung: Arbeitsintensität (47,1% Zunahme, 32,7 gleich)<br>**Beanspruchung**<br>• Zunahme physische und psychische Überlastung Mitarbeiter (63,3%)<br>• Anteile derer, die Gesundheitszustand im Vergleich zur präpandemischen Phase als schlecht (+1,5), weniger gut (+7,1%), zufriedenstellend (+8,1%) einschätzen vergrößert sich; Anteil derer mit Einschätzung gut (-9,1%) und sehr gut (-7,5%) verringert sich<br>• Anteil derer, die angeben, im Vergleich zur präpandemischen Zeit sehr oft (+3,2%), oft (+9,5%), manchmal (13,1%) krank zu arbeiten (Präsentismus) vergrößert sich<br>**Bewältigung**<br>• 62% erwarten Belastungen bewältigen zu können, 40% unsicher<br>• Bewältigungsstrategien: v.a. strukturelle Maßnahmen, Aufklärung, Stärkung Zusammenhalt, auch Inanspruchnahme finanzieller Hilfen, Vorsorgemaßnahmen, Materielle Ausstattung, technische Lösungen, Austausch und Unterstützung durch andere, Mehrarbeit
[22], 10/2020-12/2020, Deutschland	• Stresserleben, Angst, Unterstützung, Entscheidungsprozesse im Länder- und Personalkategorienvergleich<br><br>• Online-Survey<br>• N = 120 Pflegekräfte Deutschland	• Selbstentwickelter Fragebogen, 29 Items (u.a. Auswirkungen der Pandemie auf Stress-/Angst, Unterstützung innerhalb der Organisation, Externe Unterstützung für die Organisation, Lernerfahrungen, Zukunftsperspektiven)	**Beanspruchung**<br>• Stress-/Angstlevel in Großbritannien am höchsten, gefolgt von Deutschland, Schweden niedrigstes Niveau<br>**Ressourcen**<br>• Deutschland weniger interne organisationale Unterstützung (klare Leitlinien, adäquate Unterstützung durch Management, adäquate Ausrüstung mit PSA, adäquates Training) sowie externe Unterstützung (klare Richtlinien, Unterstützung durch die lokale Gemeinschaft, Unterstützung durch Familien der Klienten) als in Schweden, Großbritannien, Italien wahrgenommen
[23], 12/2020-1/2021,	• Zusammenhang pandemieassoziierte Anforderungen und Wechselabsicht	• Einzelitem für Wechselabsichten seit der Pandemiebeginn<br>• Pandemiespezifische Belastung (10 Items, u.a. Einhaltung	**Belastung**<br>• höhere Werte für pandemieassoziierte und allgemeine Belastung als in Vergleichsstichprobe<br>**Beanspruchung**

Autoren, Zeitraum, Region	Studienziel, Methode, Stichprobenumfang	Messinstrumente	Hauptergebnisse
Deutschland	• Vergleich mit Daten aus März/April 2020, kein „echter“ Längsschnitt<br>• Online-Survey<br>• N = 301 Pflegekräfte	Hygienerichtlinien, pandemieassoziierte Sorgen/Ängste, Verwendung PSA, Isolierung Patienten, Einhaltung Kontaktverbote, widersprüchliche Informationen etc.)<br>• allgemeine Belastung (12 Items, u.a. Personalmangel, Einhaltung Arbeitszeitregelungen, Arbeitsintensität, Sorge um Patientenwohl	• 20,3% Absicht, den Beruf oft /sehr oft seit Pandemie aufzugeben (Vergleichsdaten zeigen Anstieg, April: 12,8%)<br>**Zusammenhänge**<br>• Pandemieassoziierte und allgemeine Belastung assoziiert mit Berufswechselabsicht<br>• Zusammenhang zwischen allgemeiner Belastung und Wechselabsicht stärker als in Vergleichsstichprobe<br>• Kein Einfluss von Alter, Geschlecht, Einsatz in Patientenversorgung, Status, Anzahl Patienten, Organisationstyp auf Wechselabsicht
[24], 10/2022-1/2023, Bayern	• Facetten von Arbeitszufriedenheit<br>• Bleibewilligkeit/Wechselabsichten<br>• Einflussfaktoren auf Bleibeabsicht/Wechselabsicht<br>• Verbesserungsvorschläge<br>• Online-Survey<br>• N = 2572	• Adaptierte Skalen aus: IWS, MSQ, Work-Life-Balance-Scale, Nurse´s Retention Index<br>• Einzelitems Karriereentwicklung, soziale Bedürfnisse<br>• Offene Fragen Arbeitssituation, Jobwechsel	**Belastung**<br>• 50-60% unzufrieden mit: Mitbestimmung, Entlohnung, Vereinbarkeit von Beruf/Familie, Zeit für Patientenversorgung, Dienstorganisation, Pflegedokumentation<br>• weitere bedeutsame Faktoren, u.a. Personalmangel, Arbeitsüberlast, Gratifikationskrisen, Aggression Patienten & Gewalt, moralische Konflikte, hierarchische Stellung der Ärzte, Ausstiegsgründe: emotionale und körperliche Belastung, gesundheitliche Probleme, Burnout, fehlende Weiterbildungsmöglichkeiten, fehlende Vereinbarkeit Familie/Beruf<br>**Ressourcen**<br>• Unzufriedenheit Ressourcen: Beziehung zu Vorgesetzten (40,0 %), Unterstützung Mitarbeiter in schwierigen Situationen (37,8%), Teamzusammenhalt (21,3 %)<br>**Beanspruchung**<br>• Bleibemotivation 12 Monate: 56,8% ja/10,5% nein, 32,7% Bleibeabsicht bei besseren Arbeitsbedingungen<br>**Zusammenhänge**<br>• Zunehmendes Alter, Alleinleibend, höhere vertraglich vereinbarte Arbeitszeit, Berufswiederwahlabsicht, Zufriedenheit mit Karriere- und Weiterbildungsoptionen mit höherer Bleibemotivation assoziiert<br>• Tätigkeit in Altenpflege mit geringerer Chance auf Bleibemotivation assoziiert
[25], Frühjahr 2020, Deutschland	• Berufliche Anforderungen in der Pandemie<br>• Einflussfaktoren auf Rückkehrentscheidungen in den Beruf<br>• Experteninterviews		**Belastung:**<br>• Gründe für Berufsausstieg: wenig flexible Vertragsgestaltung, Arbeitsbedingungen (Fachkräftemangel, Arbeitszeiten, Abrufbarkeit, Verdienst), geringe Wertschätzung, Fehlen gewerkschaftlicher Unterstützung, fehlende

Autoren, Zeitraum, Region	Studienziel, Methode, Stichprobenumfang	Messinstrumente	Hauptergebnisse
	• N = 15		Supervision, Schwangerschaft/Elternzeit, fehlende Weiterentwicklungsmöglichkeiten<br>• Erleben der Rückkehr in den Pflegeberuf: überwiegend positiv, Reaktionen der Kollegen: Zuspruch, Unverständnis, Feindseligkeit gegenüber akademisierten Pflegefachpersonen, Rückkehr in frühere Häuser barrierearm, Barrieren bei Rückkehr in freie Häuser: Informationen zu Personalbedarf, Bewerbungsmodalitäten, Ansprechpartner, steuerliche Fragen, Bevorzugung Ehrenamtlicher aus Kostengründen<br>**Bewältigung erneuter Berufseinstieg**<br>• erhöhte Anforderungen erwartet für: Selbst-/Fremdschutz, professionelle Ausbildung, maximale Flexibilität<br>• Erwartung moralischer Konflikte: Agieren ohne ausreichende Einarbeitung, Einschränkungen der Versorgungsqualität/Equipmentmangel inklusive Versorgungsfehlern mit Todesfällen, Versorgung größerer und beatmeter Patientengruppen ohne ausreichende Schutzkleidung<br>• Erwartung psychischer und physischer Belastung (Masken, PSA, Überstunden, fehlende Familienzeit, Betreuung von Patienten, die keinen Besuch erhalten dürfen, mangelnde Wertschätzung, Umgang mit unerfahrenem, zuarbeitendem Personal, Gefahr der eigenen Ansteckung und der Ansteckung Familie)
**Setting stationäre Langzeitpflege/Pflegeheim**			
[26], 10/2020-2/2021, Deutschland	• Ursachen, Folgen von Moral Distress<br>Qualitativer Websurvey<br>N = 510		**Belastung**<br>• Personal-/Zeitmangel führen zu Rationalisierung, Priorisierung von Pflege; adäquate Pflege nicht leistbar<br>• Verschärfung Situation durch pandemiebedingte Personalausfälle, Arbeitsveränderungen, Arbeitsverdichtung, fachfremde Aufgaben in der Pandemie<br>• Leiden/Vereinsamung Bewohner durch Besuchsverbot, Kontaktreduzierung, PSA, Zeitmangel<br>• Versterben der Bewohner ohne Verabschiedung/Begleitung durch Angehörige<br>• Konflikte mit Angehörigen, Bewohner durch Auflagenwechsel, widersprüchliche Anordnungen, Verschlechterung Zustand Bewohner<br>• Eingeschränkte inter- und intraprofessionelle Zusammenarbeit und hausärztliche Versorgung sowie Symptomeskalation bis Klinikeinweisung und Infragestellen pflegerischer Expertise, Depriorisierung nicht infizierter Bewohner,

Autoren, Zeitraum, Region	Studienziel, Methode, Stichprobenumfang	Messinstrumente	Hauptergebnisse
			• Konflikte im Team, nachlassender Zusammenhalt, Respektlosigkeit und fehlende Unterstützung durch Vorgesetzte<br>• Dilemma zwischen persönlicher Sicherheit, Sicherheit der Familie und beruflicher Integrität und Verantwortung (unzureichende PSA, Aussetzen Arbeitsschutzbestimmungen, Angst vor COVID-Ausbruch auf Station, fehlende Möglichkeiten Pandemiebestimmungen durchgängig aufrecht zu erhalten)<br>• Widerspruch von Infektionsschutz und guter Pflege, Dehumanisierung der Arbeit<br>**Beanspruchung**<br>• moralischer Stress, anhaltendes Stresserleben<br>• Verlust von Authentizität, Deprofessionalisierung (S. 61)<br>• Hilflosigkeit, Angst um (Patienten-)Sicherheit<br>• Trauer, Wut, Frustration, emotionale Distanzierung, Schmerz, Müdigkeit, Schlafstörungen, reduzierte Selbstsorge, Motivationsprobleme, Erschöpfung Depressionsrisiko, Wechselabsichten
[27], 6/2020-7/2020, Deutschland	• Entscheidungssituationen und -Konsequenzen von Leitungspersonen<br>• Interviewstudie<br>• N = 78		**Belastung/Entscheidungssituationen**<br>• Entscheidungen zu sozialer Teilhabe für Bewohner/Angehörige und fehlende Handlungsgrundlagen (z.B. in palliativen Situationen, Gruppenaktivitäten)<br>• Schwierigkeit, Maßnahmen durchzusetzen bei Bewohnern mit kognitiven Einschränkungen<br>• Umsetzung von Quarantäne bzw. Isolation (Raumnutzung, Umzug/Verlegung innerhalb Einrichtung, Einzug/Rückkehr von Bewohner)<br>• Anpassung Personaleinsatz (ggf. täglich neu, erhöhter Arbeitsumfang der Leitungspersonen, Einzug von Mitarbeitenden in die Einrichtung)
[28], 10/2019-11/2019, 4/2022-6/2022, Nordrhein-Westfalen	• Psychosoziale Belastung in der palliativen Versorgung<br>• Vergleichsdaten aus Querschnitt 2019<br>• Befragung<br>• N = 55	• COPSOQ	**Belastung**<br>• tendenziell 2022 weniger Workload, weniger Jobunsicherheit bzw. Unsicherheit über Arbeitsbedingungen als 2019<br>• tendenziell 2022 mehr Work-Life-Konflikte, mehr Rollenkonflikte, weniger Rollenklarheit als 2019<br>**Beanspruchung**<br>• tendenziell 2022 weniger Präsentismus als 2019<br>**Ressourcen**<br>• tendenziell 2022 mehr Freizeit, mehr Einflussmöglichkeiten als 2019

Autoren, Zeitraum, Region	Studienziel, Methode, Stichprobenumfang	Messinstrumente	Hauptergebnisse
[29], 03/2021-06/2021, Rheinland-Pfalz	• pandemiebedingte Veränderungen<br>• Bewältigung/Maßnahmen<br>• Teilstandardisierte Leitfadeninterviews<br>• N = 10	•	**Belastung**<br>• Arbeitsintensität steigt bei vorbestehender und zunehmender Personalknappheit (z.B. durch Infektionen)<br>• Arbeitsorganisation und -inhalt verändert, Zeitdruck(z.B. emotionale Begleitung Bewohner, Ersatz für Angehörige, externe Dienste, Kontrolle Vitalparameter, organisatorische Veränderungen)<br>• häufig wechselnde Richtlinien und Vorschriften<br>• Ausbrüche, schwere Krankheitsverläufe, übermäßige Todesfälle, Leiden (Hilflosigkeit, Kontrollverlusterleben)<br>• Angst vor Ansteckung und Übertragung<br>• PSA: beeinträchtigte Atmung, Hitzestress, Kommunikation erschwert<br>• Demenzkranke Bewohner: Unverständnis/Vergessen der Hygienemaßnahmen, Übertragungsgefahr, 1:1-Betreuung wenn möglich)<br>• Konflikte mit Angehörigen: Umsetzung/Einsicht Richtlinien<br>**Bewältigung – interne Maßnahmen**<br>• ad hoc, wenig geeignet Stresserleben zu reduzieren<br>• Schulungsprogramme (Sicherheit), palliative Unterstützung hilfreich<br>• Infrastrukturelle Veränderungen (z.B. Einrichtung von Quarantänestationen)<br>• Interne Unterstützung (pflegerische Tätigkeiten von Hauswirtschaftlern)<br>• Motivierende Maßnahmen (E-Mails, Geschenkkörbe, finanzielle Anreize)<br>• Team: verbesserter Teamzusammenhalt<br>**Bewältigung – externe Maßnahmen**<br>• Personelle Unterstützung (Bundeswehr, Freiwillige, Pflegekräfte im Ruhestand)<br>• Finanzielle Wertschätzung und motivierende Maßnahmen (Applaus wenig hilfreich, Stagnation öffentlicher Wertschätzung nach erster Welle)
[30], 6/2020-7/2020, Deutschland	• Gewalt in stationären Altenpflegeeinrichtungen aus Sicht von Leitungskräften<br>• Strategien der Gewaltprävention<br>• Semistrukturierte, leitfadengestützte Telefoninterviews<br>• N = 78		**Belastung**<br>• uneinheitliche Wahrnehmung des Gewaltgeschehens<br>• nachlassendes Verständnis für Maßnahmen von Bewohnern/Angehörigen in Abhängigkeit der Umsetzung der Kontaktbeschränkungen und des Findens von Ausgleich<br>• verbale Gewalt durch Angehörige, Bewohnern aufgrund von Unverständnis, emotionaler Belastung durch Isolation;<br>• verbale Gewalt durch Mitarbeitende, aber Gefühl stärkerer Verbundenheit

Autoren, Zeitraum, Region	Studienziel, Methode, Stichprobenumfang	Messinstrumente	Hauptergebnisse
			**Bewältigung**<br>• Gewaltprävention (wenig präsent, Aufmerksamkeit auf Infektionsschutz, keine Schulungsmöglichkeit, informierte Pflegende mehr senisibilisiert)
[31], 11/2020-2/2021, Deutschland	• Stress, Angst, Depressivität<br>• Zusammenhang Einrichtungsmerkmale, personale Merkmale, Arbeitsbelastung, Stresserleben, Angst, Depressivität<br>• Online-Survey<br>• N = 811	• CoPaQ<br>• Einrichtungsmerkmale<br>• Veränderung der Arbeitsbelastung seit Pandemiebeginn<br>• Zufriedenheit mit Pandemiemanagement der Einrichtung<br>• Belastungsausmaß COVID-19-bezogener Anforderungen<br>• COVID-19-bezogene Ängste und Sorgen<br>• SDASS-21<br>• GAD-2<br>• PHQ-2<br>• COPSOQ<br>• Unterstützung am Arbeitsplatz, Feedback, Gemeinschaftssinn	**Belastung**<br>• 94,2 % starke Steigerung der Arbeitsbelastung seit Pandemiebeginn (3,8% keine Veränderung, 1,8% Abnahme)<br>• 42,3 % eher/sehr unzufrieden mit dem COVID-19-Management Einrichtungsleitung (57,7% zufrieden)<br>**Beanspruchung**<br>• 39,2 % mäßig/extrem schwere Stresssymptome<br>• 36,5% klinisch relevante Symptome von Angst<br>• 41,4% klinisch relevante Symptome von Depression<br>• 59,1 % auffällige Werte für mind. 1 Beanspruchungsfolge<br>**Zusammenhänge (Einzelitems)**<br>• COVID-bezogene Arbeitsbelastung bei Pflegefachkräften als bei Pflegehelfern, bei mehr Unzufriedenheit mit Einrichtungsmanagement, bei COVID-19-bezogenen Ängsten/Sorgen, bei Demenz-spezialisierten Pflegeheimen<br>• Unterstützung in der Arbeit mit weniger COVID-bezogener Arbeitsbelastung verbunden<br>• Stresserleben geringer in größeren Einrichtungen, höher bei höherer COVID-19-bezogener Arbeitsbelastung, COVID-19-bezogener Angst und mehr COVID-19 -Fällen unter den Bewohnern<br>• Angst höher bei höherem Belastungserleben durch COVID-bezogene Anforderungen, mehr COVID-bezogenen Ängsten/Sorgen, weniger Unterstützung am Arbeitsplatz, weniger Gemeinschaftsgefühl<br>• Depressivität höher bei Altenpflegern im Vergleich zu examinierten Pflegekräften, höher bei COVID-19-bezogenen Belastungen am Arbeitsplatz, COVID-19-bezogenen Ängsten, geringer bei Unterstützung am Arbeitsplatz und Gemeinschaftsgefühl<br>• Frauen höhere Stresslevel als Männer
[32], 6/2021, Berlin	• Belastungen, Bewältigungsstrategien, Entlastungsangebote, Ressourcen vor und während der ersten Welle in der COVID-19-Pandemie		**Belastung und Bewältigung vor Pandemie**<br>• Belastungsfaktoren: Personalmangel, Zeitdruck, Arbeitsdichte, Krankenstand; fehlende Sichtbarmachung und Aufarbeitung von Belastungen, Gewalt, Zunahme Dokumentationsaufwand, Schichtdienst

Autoren, Zeitraum, Region	Studienziel, Methode, Stichprobenumfang	Messinstrumente	Hauptergebnisse
	• Mixed Methods (nur Interviews berücksichtigt)<br>• N = 18		• Bewältigungsstrategien: Lesen von Büchern, Gespräche im Privaten, Überlastungsanzeigen nicht erfolgreich/eingestellt, Reduzierung Arbeitsaufgaben – Senkung Pflegequalität<br>**Belastung und Bewältigung zu Pandemiebeginn**<br>• Reaktion auf unvorhersehbare Anforderungen, kein geregelter Tagesablauf, wechselnde Empfehlungen, fehlendes Fachwissen, Erfahrungen, Struktur<br>• Personalnotstand, weitere Personalausfälle durch Infektion/Quarantäne<br>• Mehrarbeit (z. B. durch zusätzliche Wege zur Pforte Geschenke für Bewohner abholen, Telefonanrufe, Durchführung von Schnelltests)<br>• Starke Belastung durch ausgeprägte Symptomatik, Todesfälle bei Bewohnern (z.B. Beistand/Hilflosigkeit bei Luftnot)<br>• Kontaktverbote fördern Einsamkeit, Irritation, Leid, aggressives Verhalten der Bewohner, Verzweiflung und verbale Angriffe bis körperliche Gewalt bei Angehörigen<br>• Mangel an PSA und Desinfektionsmittel, Angst vor Übertragung größer als vor eigener Infektion<br>• Körperliche Belastung durch Maskentragen, Fehlende Pausen<br>• hohe moralische Belastung, wenn keine Sterbebegleitung möglich<br>• Private Belastung: Kompensation von KITA- und Schulschließungen<br>• Ab 2021 (Impfung, Kontaktmöglichkeiten) „annähernd normaler Alltag“ (S. 326)<br>**Beanspruchung**<br>• Erschöpfung & Kreislaufprobleme durch PSA, Verunsicherung hinsichtlich Virusübertragung, emotionale Erschöpfung, Traurigkeit, Müdigkeit<br>**Bewältigung**<br>• Keine Fallbesprechungen oder Supervision<br>• Z.T. Unterstützung, Dank, Wertschätzung von Leitung, Führungskraft, Träger<br>• Leasingkräfte nicht hilfreich (unvorbereitet, Sprachbarriere)<br>• Unterstützung durch Bundeswehr (Testungen) hilfreich<br>• Stärkung Teamzusammenhalt, Unterstützung, Wertschätzung, Solidarität, digitale Kommunikation<br>• Entspannung/Stressbewältigung: Radfahren, Gespräche, Lesen, Garten-/Grundstücksarbeit

Autoren, Zeitraum, Region	Studienziel, Methode, Stichprobenumfang	Messinstrumente	Hauptergebnisse
			• Bleibefaktoren: Teamarbeit, Interaktion mit Pflegebedürftigen, Sinnhaftigkeit, Gratifikation (Freude über Bonuszahlungen)<br>• Annahme: Wertschätzung durch Gesellschaft, Politik nicht von Dauer<br>• Wünsche: mehr Personal, höherer Personalschlüssel
[33], 4/2020-5/2020, Deutschland	• Virusausbreitung<br>• Personelle & materielle Ressourcen<br>• Organisatorische Maßnahmen<br>• Unterstützungswünsche<br>Online-Survey<br>N = 824	• Eigens entwickelte Items (Einrichtungsmerkmale, Vorkommen SARS-CoV-2-Virus, personelle & sachliche Ausstattung, Kommunikationsstrukturen, Wünsche an Politik)	**Belastung**<br>• 71% COVID-19-bezogene Personalausfälle<br>• 75% zeitliche Mehraufwände durch Hygiene-/Infektionsschutzmaßnahmen, Wegfall Ehrenamtlicher (ca. 61,7min pro Schicht/Pflegekraft)<br>• PSA-Mangel größer als Mangel Desinfektionsmitteln; zum Erhebungszeitpunkt zahlreiche Engpässe behoben<br>**Bewältigung**<br>• 98,7% Kontaktreduktion, z.B. Aussetzen von Teamsitzungen<br>• 61,7% im Vorfeld nicht auf Pandemie vorbereitet, 88,9% kein Thema in Ausbildung, 95,1% kein Thema im Studium, 72% kein Thema in Fortbildung<br>• 90,7% Schulungen zu Schutzbekleidung, Hygieneeinweisungen, Umgang mit erkrankten Bewohnern<br>• 97% Abwesenheitsregelungen für infizierte Mitarbeitende<br>• Personalmanagement: 39,5% keine Maßnahmen notwendig, 33,1% Umverteilung Stammpersonal, 28,5% Stundenaufstockung, 16,3% Zeit- und Leiharbeiter, Initiative #pflegereserve ohne Bedeutung<br>**Wünsche**<br>• 85,4%-64% Corona-Prämie für Pflegekräfte, Prämie für Berufsrückkehrer
[34], 5/2020-7/2020, Deutschland	• Herausforderungen, Strategien, Konsequenzen auf Organisationsebene, für Versorgung<br>• halbstrukturierte Telefoninterviews<br>• N = 78		**Belastung organisationale Ebene**<br>• hoher Workload Krisenstab (wechselnde Regelungen, Personalinformation<br>• höherer Personalbedarf durch zusätzliche Aufgaben (z.B. Umsetzung Kontaktbeschränkungen, Bedürfnisbefriedigung Angehörige)<br>• höhere kommunikative Anforderungen (z.B. Videoanrufe für Bewohner/Angehörige, Anschaffung Technik und Geräteeinweisung; Missverständnisse/erhöhter Kommunikationsbedarf mit anderen Versorgungssektoren aufgrund fehlenden persönlichen Kontakts)<br>**Bewältigung organisationale Ebene**<br>• Krisenstab, Reorganisation Gebäude-/Raumnutzung, Anpassung/Umsetzung Hygieneplänen, Nutzung informeller Netzwerke für

Autoren, Zeitraum, Region	Studienziel, Methode, Stichprobenumfang	Messinstrumente	Hauptergebnisse
			Materialbeschaffung, Anpassung Personaleinsatz (u.a. Aufstockung Nachtpersonal, Einsatz von Mitarbeitenden aus Kindertagesstätten, Restaurants, Leiharbeit)<br>**Belastung Pflegebereich**<br>• mehr Zeit-/Personalaufwand durch veränderte Routinen (z. B. Essensausgabe)<br>• Übernahme nicht-pflegerischer Aufgaben (Begleitung/Einweisung Besucher, Testungen, Fußpflege, Friseur, Gymnastik, Desinfektion)<br>• erhöhte medizinische Verantwortung (weniger/kein Besuch/Unterstützung von Medizinern, Gefühl Kompetenz-/Gesetzüberschreitung)<br>• erhöhte Dokumentationsanforderungen (Vitalzeichen, Symptome, Besucher)<br>• Förderung sozialer Teilhabe (z. B. Briefeschreiben, Vorlesen von Briefen)<br>• erhöhte Kommunikationsanforderungen (Einzelgespräche mit Bewohnern, behinderte Kommunikation durch PSA, veränderte Übergabestruktur führte zu Mehraufwand/Informationslücken)<br>• Überstunden<br>**Beanspruchung Mitarbeiter**<br>• Stresserleben und höheres Konfliktpozential mit kognitiv eingeschränkten Bewohnern/Angehörigen<br>• Angst/Überforderung aufgrund fehlender PSA, Übertragungsgefahr, reduzierter medizinischer Versorgung und gestiegener Verantwortung<br>**Ressourcen**<br>• neu entstandener Teamzusammenhalt<br>• ruhigere Atmosphäre, ganzheitliche Pflege<br>• stärkeres Verbundenheitsgefühl Pflegepersonal und Bewohner/innen<br>• Gewöhnungseffekt nach erster Welle
[35], 8/2020-10/2020, Interviews 6/2021, Brandenburg	• Psychosoziale Belastung<br>• positive Effekte der Pandemie<br>• Vergleich mit präpandemischen Vergleichsstichprobendaten<br>• Online-Survey, Semistrukturierte Interviews<br>• N = 177 (Befragung) und N = 15 (Interviews)	• Befragung: COPSOQ, PSS	**Belastung im Vergleich zu präpandemischen Vergleichsdaten**<br>• Höhere Werte für Emotionsarbeit/Emotionen verbergen, Konflikte Arbeit-Privatleben, Rollenkonflikte, physische Belastung, Workload<br>**Beanspruchung im Vergleich mit präpandemischen Vergleichsdaten**<br>• Höhere Werte für Burnout, Berufswechselabsichten, Präsentismus, Unfähigkeit zur Entspannung<br>**Ressourcen im Vergleich mit präpandemischen Vergleichsdaten**<br>• Höhere Werte für Bedeutung der Arbeit, Menge der sozialen Beziehungen

Autoren, Zeitraum, Region	Studienziel, Methode, Stichprobenumfang	Messinstrumente	Hauptergebnisse
			• Geringere Werte für Unterstützung in der Arbeit, Qualität der Führung, Wertschätzung<br>**Interviews**<br>**Belastungskategorien:** Veränderung allgemeiner Arbeitsbedingungen, Zunahme Workload, Sorge um isolierte Bewohner, Umgang mit Angehörigen (Beschimpfung, Information, Testung), Übernahme der emotionalen/sozialen Begleitung der Bewohner; Fehlende Möglichkeit zur Sterbebegleitung, Infektions-/Übertragungsangst, Stigmatisierung, Schulgefühle bei positiver Testung, Technisierung der Pflege durch strikte Einhaltung von Hygieneprotokollen, Kommunikationsbarrieren<br>**Ressourcen:** stärkerer Zusammenhalt (zu Beginn z.T. auch Misstrauen, befürchtete Fluktuation konnte nicht beobachtet werden)
[36], 8/2020-12/2020, Brandenburg	• psychosoziale Belastung<br>• Zusammenhang zwischen COVID-19-bezogenen Sorgen/Ängsten und Burnout<br>• Befragung (Paper-Pencil)<br>N = 195	• COPSOQ (Erschöpfung)<br>• Corona-Fragebogen (ReCoDe-Studie)<br>• PSS-10	**Belastung:**<br>• Sorge Eigeninfektion: 16,55 äußerst/sehr, 22,1% mäßig<br>• Sorge Übertragung auf Pflegebedürftige: 28,2% äußerst/sehr, 17,9% mäßig<br>• Sorge Übertragung auf Freunde/Familie: 28,8% äußerst/sehr, 14,4% mäßig<br>**Beanspruchung:**<br>• 56,3% immer/oft, 31,8% manchmal körperlich erschöpft, 40% immer/oft emotional erschöpft, 38,5% manchmal, 48,2% immer/oft, 32,3% manchmal ausgelaugt<br>**Zusammenhänge**<br>• mehr Heimplätze (>50), Sorge vor Infektion/Übertragung mit höheren Burnoutwerten verbunden
**Setting Klinik**			
[37], 11/2020-12/2020, Deutschland	• Testung Hypothesen zur Job-Demands-Resources-Theory (Fokus motivationale Schiene, Engagement)<br>• Online-Survey<br>• N = 1027	• COPSOQ<br>• GTL<br>• professionelle Schlüsselressourcen (Schutzausrüstung, Zugang zu Information, klare Anforderungen)<br>• UWES	**Zusammenhang Arbeitsbelastung, Ressourcen, Beanspruchung**<br>• 36% Varianzaufklärung Arbeitsengagement<br>• Autonomie, professionelle Ressourcen, zwischenmenschliche Beziehungen positiv mit Engagement assoziiert<br>• Geringe Belohnungen negativ mit Engagement assoziiert<br>• Arbeitsüberlastung positiv mit Arbeitsengagement assoziiert<br>• kein Einfluss von transparentem Management, Work-Life-Interferenzen, transformationaler Führung

Autoren, Zeitraum, Region	Studienziel, Methode, Stichprobenumfang	Messinstrumente	Hauptergebnisse
[38], 4/2020, Deutschland	• Wahrnehmung der Arbeitssituation von Pflegenden auf Intensivstationen, Konsequenzen für Versorgung<br>• Webbasierter qualitativer Survey<br>• N = 397		**Belastung**<br>• Extremer Personalmangel (Dienstverpflichtungen, Arbeitszeitverlängerungen, mehr Patienten versorgen, Steigerung Belastung, andere Stationen Langeweile, Unterforderung)<br>• Warten (Ungleichverteilung/Steuerung von Ressourcen)<br>• PSA (Mangel, Wiederaufbereitung, Reduzierung Quarantäne, gesundheitliche Beeinträchtigungen, Grundbedürfnisse nicht stillen können)<br>• Lernen, schlechte Informationen, fehlende Regelungen<br>• Versorgung: ungewohnte Routinen/Prozeduren, emotionale Belastung durch Tod/Sterben, ethische Belastung Sterbebegleitung, Besuchsverbote für Angehörige gleichzeitig Verkürzung Quarantäne des Personals)<br>• Kompensationsversuche (Improvisation, Patientengefährdung, Übernahme fachfremder Aufgaben)<br>**Beanspruchung**<br>• ungutes Gefühl, psychische Erschöpfung, Angst vor Infektion/Übertragung
[39], 4/2020-7/2020, Deutschland	• Verausgabungs-Belohnungsungleichgewichte in Abhängigkeit von Geschlecht, Beruf<br>• Einflussfaktoren auf Verausgabung, Belohnung<br>• Online-Survey<br>• N = 923	• Einzelitems Personalknappheit, Erholungszeit, Vertrauen in Kollegen, Infektions-/Übertragungsangst, Sicherheitsgefühl<br>• Veränderungen des Stresserlebens vor/nach der Pandemie<br>• ERI<br>• Optimismus (1 Item),<br>• ESSI, SOC Scale	**Belastung, Ressourcen**<br>• 14,1% Wechsel der Abteilung<br>• Höhere Verausgabungswerte als andere Berufsgruppen (außer Verwaltung)<br>• geringe Gratifikationswerte als andere Berufsgruppen (MTA nicht signifikant)<br>• 62,8% Verausgabungs-/Belohnungsungleichgewicht (MTA: 58,8%, Mediziner: 41,8%)<br>• Pflegekräfte 2fach höhere Chance für Verausgabungs-Belohnungsungleichgewichte im Vergleich zu Medizinern<br>**Beanspruchung**<br>• Prädiktoren für ERI-Ungleichgewicht in Gesamtstichprobe: unzureichendes Personal, unzureichende Erholung, fehlendes Schutzgefühl durch Arbeitgebermaßnahmen, hohe Belegungsrate, unzureichendes Vertrauen in Kollegen, Pflegeberuf<br>• Frauen (51,8%) 1,175 höhere Chance als Männer (47,8%) für ERI-Ungleichgewicht; erleben weniger Belohnung als Männer
[40], 4/2020-7/2020, München	• Endocannabinoide, Endocannabionoid-ähnliche Marker, Kortisonkonzentration im Haar zu 4 Messzeitpunkten	• Haaranalysen<br>• CoPaQ<br>• BRS<br>• Einhaltung COVID-Regeln	**Beanspruchung**<br>• Pflegekräfte berichten weniger Compliance für COVID-Maßnahmen als Mediziner

Autoren, Zeitraum, Region	Studienziel, Methode, Stichprobenumfang	Messinstrumente	Hauptergebnisse
	• psychische Belastung, Resilienz<br>• Mixed-Methods<br>• N = 85		• Belastung auf Endoskopie-/Notfallstationen geringer als auf den Normalstationen<br>• Höhere Kortisonkonzentrationen bei Pflegenden im Vergleich zu Medizinern zu t1 und t3, keine Unterschiede in subjektiven Stressleveln
[41], 11/2020-12/2020, Deutschland	• pflegespezifische Anforderungen, Ressourcen, Arbeitsengagement<br>• Online-Survey<br>• N = 1027	• COPSOQ (gekürzt, Arbeitsüberlastung, Mangel an Belohnung, Work-Life-Interferenzen, Autonomie, zwischenmenschliche Beziehungen, faires, authentisches Management)<br>• Unterstützung von Vorgesetzten<br>• professionelle Ressourcen<br>• UWES-9<br>• Global Transformational Leadership Scale	**Belastung**<br>• Mehr Arbeitsüberlastung, weniger Work-Life-Interferenzen im Vergleich mit präpandemischen Referenzwerten<br>• ambulante Pflege weniger Arbeitsüberlastung, Work-Life-Interferenzen, Mangel an formaler Belohnung als stationäre Altenpflegeeinrichtungen, Kliniken<br>• Pflegende in COVID-Versorgung höhere Arbeitsüberlastung, Work-Life-Interferenzen, Mangel an formalen Belohnungen<br>**Beanspruchung**<br>• Arbeitsengagement geringer als in Normstichprobe<br>• Pflegende mit Kontakt zu COVID-Patienten weniger engagiert als Pflegende ohne Kontakt<br>**Ressourcen**<br>• Soziale Beziehungen, Autonomie im Vergleich zu präpandemischen Referenzwerten günstiger<br>• Mangel an formaler Belohnung geringer in ambulanter Pflege als in stationären Altenpflegeeinrichtungen, Kliniken<br>• Ressourcenausstattung in ambulanter Pflege günstiger als in stationären Altenpflegeeinrichtungen, Kliniken<br>• Pflegende aus COVID-Bereichen höhere Werte für professionelle Ressourcen, faires, authentisches Management, Unterstützung durch Vorgesetzte, transformationale Führung, Autonomie als Pflegende in NON-COVID-Bereichen
[42], 4/2020; 8/2020, Hamburg	• Seroprävalenz Sars-Cov-2 zwischen t0 und t1<br>• somatische Symptome<br>• Befragung<br>• N = 1792	• SSBS, SSD-B<br>• PHQ-4<br>• Erschöpfung/Fatigue (NRS, 1 Item)<br>• Einsamkeit (NRS, 1 Item)<br>• COVID-19-Angst (NRS, 1 Item)	• Beruf Pflege positiv korreliert mit körperlichen Symptome t1

Autoren, Zeitraum, Region	Studienziel, Methode, Stichprobenumfang	Messinstrumente	Hauptergebnisse
[43],<br>10/2020-6/2021,<br>Deutschland	• Entscheidungskonflikte/-unsicherheit<br>• N = 369	• Im Delphiverfahren entwickelte Skalen zu Entscheidungskonflikten/-unsicherheit	**Belastung**<br>• Veränderung der klinischen Entscheidungssituation in der Pandemie: >25% der Pflegekräfte Onkologie; > 50% der Pflegekräfte Psychiatrie<br>• Pflegekräfte stärker durch Entscheidungskonflikte belastet als Mediziner<br>• Pflegekräfte Onkologie stärkere Belastung durch Entscheidungskonflikte, Pflegekräfte Psychiatrie höchste Werte Entscheidungsunsicherheit
[44],<br>11/2020-3/2021,<br>Deutschland	• Validierung der 9-Item-Skala Stress und Angst vor Epidemien, Validierungsskalen: Angst, Depression, Schlaflosigkeit<br>• Online-Survey<br>• N = 241	• SAVE-9 (Subskalen COVID-Angst, Veränderungen der Arbeit durch Pandemie)<br>• GAD-7<br>• PHQ-9<br>• ISI	**Beanspruchung**<br>• COVID-bezogene Angst bei Pflegekräften höher als in Gesamtstichprobe<br>• milde Ausprägung für Angst, Depression, kein Anhalt für Schlafstörungen<br>• Pflege höhere Werte als Mediziner für alle Variablen<br>• höhere Werte für Covid-bezogene Angst, Angst (GAD) als Vergleichswerte Welle 1 aus anderen Ländern (z.B. Russland, Korea)
[45],<br>4/2020-5/2020,<br>Deutschland	• Subjektive Belastung<br>• Informationspolitik in Pandemie<br>• Angemessenheit struktureller Maßnahmen<br>• Online-Survey<br>• N = 1317	• Einzelitems zu arbeitsbezogenen Stressoren (z. B. Veränderungen Workload), personbezogenen Stressoren (Angst vor Infektion/Übertragung des Virus), Ressourcen (z.B. Wertschätzung durch Arbeitgeber), Beanspruchungsfolgen (z.B. Mentale Beanspruchung, Schlafqualität, Wechselabsichten)	**Belastung**<br>• Pflege höhere arbeitsbezogene/personenbezogene Belastung (Workload, weniger Erholungszeit, Sorgen über Zukunft/Familie, Angst vor Ansteckung/Übertragung) als andere Personalgruppen<br>**Beanspruchung**<br>• Beanspruchung höher (mental, Stresserleben, weniger Arbeitszufriedenheit, weniger Schlafqualität, mehr Zustimmung zu Wechselabsichten) als andere Personalgruppen<br>**Ressourcen**<br>• Pflege weniger Wertschätzung durch Arbeitgeber, weniger gut informiert durch Vorgesetzte, erleben Kliniken weniger gut vorbereitet auf Pandemie als andere Berufsgruppen
[46],<br>11/2020-2/2021,<br>Hannover	• wahrgenommene Risiken, Schutz- und Präventionsmaßnahmen in der Pandemie<br>• Online-Survey<br>• N = 460	• Infektionsrisiko, Infektionsangst, tatsächliche Infektion<br>• Schutz-/Präventionsmaßnahmen<br>• Einhaltung Schutz-/Präventionsmaßnahmen im privaten Bereich	**Belastung**<br>• Pflege schätzt Risiko, infiziert worden zu sein, höher als Mediziner ein<br>• Pflege mehr Infektionsangst im privaten Bereich als Mediziner<br>• Gesamtstichprobe: Risiko für Infektion 1 %, Risikoeinschätzung Personal 15 %, höhere Infektionsangst bei Kontakt mit COVID-19-Patienten
[47],<br>12/2020-1/2021,<br>Hessen	• Einfluss von arbeitsbezogenem SOC, organisationaler Unterstützung auf Burnout<br>• Zusammenhang Burnout und Wechselabsichten	• Einzelitems physische, psychosoziale Stressoren, Unterstützung Arbeitgeber, Politik<br>• BAT<br>• Work-SOC<br>• POS-s	**Beanspruchung**<br>• 25,4% moderates, 22,2% hohes Burnoutlevel, Mittelwert über Durchschnitt Beschäftigter in Deutschland<br>• 20,7 % Arbeitsplatzwechsel nach der Pandemie<br>• Pflegende in COVID-19-Versorgungsbereichen höhere Burnoutmaße, höherer Anteil Wechselabsichten

Autoren, Zeitraum, Region	Studienziel, Methode, Stichprobenumfang	Messinstrumente	Hauptergebnisse
	• Einflussfaktoren auf SOC und organisationale Unterstützung<br>• Online-Survey<br>• N = 595		**Ressourcen**<br>• Schulungsangebote von 24,7 % als positiver Faktor genannt, Wunsch: Achtsamkeits- und Resilienztraining, Angebote zur Kinderbetreuung, zu gesundheitsbezogenen Themen<br>• 55,1 % unzureichende Unterstützung durch Arbeitgeber<br>**Zusammenhänge**<br>• negativer Zusammenhang zwischen arbeitsbezogenem SOC sowie Unterstützung durch Organisation und Burnout<br>• positiver Zusammenhang zwischen Burnout und Wechselabsicht<br>• SOC höher bei Schulungsangeboten, höheres Alter, Wahrnehmung durch Mehrarbeit Beitrag zur Pandemie zu leisten, Patienten fachlich versorgen können
[48], 4/2020-7/2020, Deutschland	• Depression, Angst<br>• Arbeitsbezogene und pandemieassoziierte Stressoren<br>• Einflussfaktoren auf Depressions- und Angstsymptome<br>• Online-Survey<br>• N = 1275	• Personalausstattung, Workload, PSA, Belegung, Kontakt zu COVID-Patienten/-Material, Versetzung, COVID-19-Sorge/Angst, Sicherheitsgefühl, Erholungsmöglichkeiten, Vertrauen/Verlässlichkeit im Team,<br>• PHQ4<br>• Schlaf, Erschöpfung<br>• Rauchen, Alkoholkonsum, Einnahme Antidepressiva/Beruhigungsmittel	**Belastung**<br>• 34,5% hohe/leicht überdurchschnittliche Belegungsrate<br>• 18,5% versetzt in der Pandemie<br>• Personalausstattung seltener als von Medizinern, MTA ausreichend eingeschätzt<br>**Beanspruchung**<br>• 21,6% auffällige Werte Depressivität, 19% Angst<br>• Höhere Werte für Depression/Angst im Vergleich zu dt. Normstichprobe vor Pandemie<br>• geringere Werte für Depression/Angst im Vergleich zur dt. Allgemeinbevölkerung<br>**Zusammenhänge**<br>• unzureichende Erholung, erhöhter Alkoholkonsum mit häufigeren Depressionssymptomen assoziiert<br>• Angst vor Ansteckung, unzureichende Erholung, höherer Alkoholkonsum mit häufigeren Angstsymptomen assoziiert
[49], 3/2021-4/2021, Augsburg	• Beanspruchung (Vergleich Querschnitt aus 2020, zwischen Pflege/Medizin, Kontakt zu COVID-19-Patienten)<br>• Zusammenhang Resilienz, Schlafqualität, Beanspruchung<br>• Online-Survey	• PHQ,<br>• MBI<br>• AIS<br>• CD-RISC	**Beanspruchung**<br>• Pflege höhere Werte für Burnout (Erschöpfung, Zynismus, weniger Professionelle Effizienz), Stresserleben, Angst, Depression als Medizin<br>• Kontakt mit COVID-19-Patienten positiv assoziiert mit Burnout (Erschöpfung, Zynismus)

Autoren, Zeitraum, Region	Studienziel, Methode, Stichprobenumfang	Messinstrumente	Hauptergebnisse
	• N = 152		• Anstieg Erschöpfung, Stresserleben, Angst, Depression im Vergleich Querschnittdaten derselben Klinik in Welle 1, Werte für Zynismus, Professionelle Effizienz stabil (kein echter Längsschnitt)
[50], 11/2020-7/2021, Hessen	• Führung in Pandemie<br>• Maßnahmen zur Unterstützung des Personals, Lessons learned<br>• Telefoninterviews<br>• N = 26		• Wechsel von strategischer zu fürsorglicher (gesundheitsförderlicher) Führung (= Beachtung Belastungen des Personals, Entwicklung entlastender Maßnahmen, z.B. Anpassung Arbeitszeit für Eltern)<br>• Steigerung Anforderungen zwischen Welle 1 und 2 (Personalausfälle, Impfung, Quarantäne; Versterben von Patienten jüngeren Alters, Häufung von Sterbefällen)<br>• hoher psychosozialer Unterstützungsbedarf des Personals wahrgenommen<br>• Stationsleitungen: Belastungskumulation (Versorgung/organisatorische Aufgaben)<br>• Berufserfahrung, Resilienz, Belastbarkeit, Optimismus, Ruhe/Gelassenheit Basis von Self Care
[51], 4/2020-7/2020, Deutschland	• Zusammenhang SOC, soziale Unterstützung, Religiosität, Depression, Angst<br>• Veränderung Belastung vor/in Pandemie und Zusammenhang mit SOC, sozialer Unterstützung, Religion<br>• Online-Survey<br>• N = 1171	• Veränderung Arbeitsbelastung seit Pandemiebeginn<br>• SOC-3, ESSI, TPV<br>• PHQ-2<br>• GAD-2	**Belastung**<br>• deutliche Steigerung Arbeitsbelastung<br>**Beanspruchung**<br>• Werte Depression/Angst unterhalb Werte MTA, Mediziner weniger depressiv<br>**Ressourcen**<br>• vergleichbare Werte für SOC, soziale Unterstützung wie Mediziner; höhere Werte für SOC und soziale Unterstützung als MTA<br>**Zusammenhänge**<br>• nur SOC, nicht soziale Unterstützung (Fokus Freunde/Familie) negativ mit Depressivität und Angst assoziiert<br>• hoher SOC bester Prädikor für geringer wahrgenommene Veränderung der Belastungssituation, soziale Unterstützung ohne Einfluss auf Belastungswahrnehmung<br>• Religiosität ohne bedeutsame Effekte<br>• Weibliches Geschlecht signifikant positiver Zusammenhang mit sozialer Unterstützung, Angst, Depression; negativer Zusammenhang mit SOC<br>• Höheres Alter positiv mit SOC, Religiosität und negativ mit sozialer Unterstützung und Depression assoziiert
[52], 4/2020-	• Zusammenhang Stresserleben, Angst, Depressivität	• Veränderung Arbeitsbelastung seit Pandemiebeginn	**Zusammenhänge**<br>• Keine professionsbezogenen Unterschiede in Resilienzwerten

Autoren, Zeitraum, Region	Studienziel, Methode, Stichprobenumfang	Messinstrumente	Hauptergebnisse
7/2020, Bonn	• Zusammenhang Resilienz, Angst, Depressivität<br>• Moderierende/mediierende Effekte von Resilienz<br>• Online-Survey<br>• N = 330	• RS-5<br>• PHQ-4	• Kontakt zu COVID-Patienten, Berufserfahrung, Teilzeitarbeit ohne Einfluss auf Resilienzmaße
[53], 4/2020-7/2020, Deutschland	• moralischer Stress, Stresserleben im Vergleich vor der Pandemie,<br>• Angst, Depressivität<br>• Zusammenhang zwischen Alter, Geschlecht, Berufserfahrung, Belegungsgrad, Kontakt zu Covid-19-Patienten, Anzahl der bestätigten COVID-19-Fälle, Angst, Depression, Stresslevel, Moralischer Stress<br>• Online-Survey<br>• N = 1149	• Stresserleben<br>• MDT<br>• PHQ-4	**Belastung**<br>• 26,5% mehr Belegung als Durchschnitt<br>**Beanspruchung**<br>• Moral Distress im mittleren Bereich der möglichen Ausprägungen (oberhalb der Werte Pflegender aus Onkologie, ITS vor Pandemie)<br>• Pflegekräfte höhere Moral Distress-Werte als Medizin<br>• Anstieg Stresserleben im Vergleich vor/in der Pandemie wahrgenommen<br>• klinisch relevante Werte Depressivität 21%, Angstwerte 20%
[54], 572021-7/2021, Deutschland	• Häufigkeit, Prädiktoren von Krankheitsausfällen und Kündigungsabsichten<br>• Online-Survey<br>• N = 757	• Veränderungen Workload, Personalausstattung, Versetzung, Kontakt zu COVID-19-Patienten, Sorge/Angst vor Ansteckung, Kündigungsabsicht, Absicht Arbeitszeit zu reduzieren, Schlafstörung, Vertrauen in Kollegen, Schutzmaßnahmen durch Organisation<br>• ERI<br>• WAI (1Item Krankentage),<br>• PHQ-2<br>• GAD-2	**Belastung**<br>• 60,9% Stationsbelegung etwas /stark erhöht<br>**Beanspruchung**<br>• 18,9% Absicht Arbeitsplatz im Gesundheitswesen aufzugeben, 21,9% Kündigung/Wechsel Arbeitsstelle innerhalb Gesundheitswesen<br>• 28,4% Absicht Arbeitszeitreduzierung<br>• 32,5 % Krankentage ≥ 10 Tagen/12 Monate, 2,3 % Krankentage ≥ 25 Tagen/12 Monate<br>**Zusammenhänge**<br>• Anteil Teilnehmende mit Kündigungsabsicht höher bei Alter< 50 Jahren, Berufserfahrung < 3 Jahre, SARS-CoV-2-Infektion, Unsicherheit hinsichtlich Infektion, Kontakt mit infizierten Patienten/Material, Vorerkrankungen<br>• Chance für Kündigungsabsicht höher bei Abteilungswechsel während Pandemie, höheren Depressionssymptomen, Teilzeitarbeit<br>• Höhere Belohnungsmaße mit 15% weniger Kündigungsabsicht assoziiert

Autoren, Zeitraum, Region	Studienziel, Methode, Stichprobenumfang	Messinstrumente	Hauptergebnisse
			• Anteil Teilnehmer mit ≥ 10 Krankentage höher bei Infektion mit SARS-CoV-2, Vorerkrankungen, Erschöpfung, Vertrauen in Kollegen, Angst vor Ansteckung<br>• Chance für ≥ 10 Krankentage reduziert bei Kontakt mit infizierten Patienten, Wahrnehmung ausreichender Personalausstattung, mehr Belohnung<br>• Mehr Frauen als Männer waren in den letzten 12 Monaten mehr als 10 Tage krank (33.7% vs. 28,9%); mehr Männer als Frauen beabsichtigen den Beruf zu verlassen (18.0% vs. 22,9%); aber kein Effekt von Geschlecht auf Krankentage, Kündigungsabsicht im Gesamtmodell
[55], 5/2020, Deutschland	• Pandemiestressoren<br>• Ressourcen, positive Veränderungen<br>• Telefoninterviews<br>• N = 8		**Belastung:**<br>• Bereichsspezifische Auslastung in Pandemie (30%-80%), Pandemiebeginn = Ausnahmesituation<br>• mangelnde Verfügbarkeit von Personal (Quarantäne, wegfallende Kinderbetreuung, Krankmeldungen; Arbeitsverdichtung auf Normalstationen durch Versetzung, Erkrankung), Sterben von Patienten im selben Alter, Veränderungen Hygienestandards, Isolationszimmer über 12h, Umverteilung in neue Teams, erwartete hohe Flexibilität, eingeschränkter/fehlender sozialer Kontakt durch Isolierung<br>**Beanspruchung**<br>• Pandemiebeginn Anstieg Krankmeldungen - Vermutung = Angst vor Ansteckung,<br>**Ressourcen:**<br>• Lernen von anderen Bereichen, interprofessionelle Zusammenarbeit, Zusammenhalt,<br>**Bewältigungshandeln/Lösungen:**<br>• Materialaufbereitung, Stationspriorisierung für Materialverteilung, Ausfallkonzepte, Personal-Umverteilung - schwierig bei Weigerung auf ITS zu arbeiten, Regulierung des Patientenaufkommens durch einrichtungsübergreifendes Meldesystem, Stufenpläne für Massenanfall an Patienten, Bettensperrung
[56], 3/2020, Deutschland	• Psychische Beanspruchung<br>• Online-Survey<br>• N = 1511	• Pandemiebezogene Sorgen/Angst<br>• GAD-7<br>• PHQ-2<br>• EuroQoL/EQ-5D-3L	**Belastung**<br>• Pandemieassoziierte Sorgen/Angst vergleichbar Medizin<br>• 63,7% Zugang zu Schutzausrüstung vorhanden<br>**Beanspruchung**<br>• Auffällige Angstwerte: 11,41% (Medizin: 5,89%, Rettungsdienst: 4,55%)

Autoren, Zeitraum, Region	Studienziel, Methode, Stichprobenumfang	Messinstrumente	Hauptergebnisse
			• höhere Werte Angst, Depressivität, geringere Werte gesundheitsbezogene Lebensqualität als Medizin, Rettungsdienst<br>• auffällige Angstmaße bei 14,69% mit geringem, bei 8,8% mit hohem Informationsgrad
[57], 3/2021-7/2021, Deutschland	• Zusammenhang psychische Beanspruchung, moralischer Stress<br>• Zusammenhang zwischen Arbeitsbelastung, soziodemografischen Faktoren, psychischer Beanspruchung, Moral Distress bei Pflegekräften in der Onkologie<br>• Online-Survey<br>• N = 125	• FACT-19<br>• MDT<br>• PHQ-9 (Depressivität)<br>• GAD-7 (Angst)<br>• PHQ-15 (Somatisierung)<br>• MBI	**Belastung**<br>• 73,8%: ziemliche/starke Zunahme Arbeitsbelastung seit Pandemie, 63,2% ziemlicher/starker Personalmangel seit Pandemie, 51,8% ziemlich/starke Begrenzung des Beziehungsaufbaus zu Patienten seit Pandemie<br>• 63,7% Zugang zu Schutzausrüstung vorhanden, 62,4% ziemliche/starke Zustimmung ausreichendes Schutzgefühl<br>• zunehmender Workload mit Personalmangel (80 %), Hygieneanforderungen (84,8 %), Verlust Angehörigenunterstützung (56,8 %) assoziiert<br>**Beanspruchung**<br>• mäßig schwere/schwere Depressivität 13,8% (mild: 35%), Angstsymptome 24,0% (mild: 41,3%), schwere Somatisierungssymptome 22,5% (gering: 32,4%)<br>• Burnout: 46,6% mind. für ein Burnout-Merkmal hohe Ausprägungen<br>• Hohe Maße moralischer Stress: 60,0%<br>**Zusammenhänge**<br>• moralischer Stress mit Pflegeberuf verbunden<br>• Depression mit moralischem Stress, weiblichem Geschlecht, höherem Workload verbunden<br>• ausreichendes Gefühl des Schutzes mit weniger, Burnout mit mehr moralischem Stress verbunden
[58], 4/2020-7/2020, Deutschland	• PTBS<br>• Zusammenhang zwischen PTBS, psychosoziale Gesundheit<br>• Online-Survey<br>• N = 1277	• Kontakt mit infizierten Patienten/ Material, Versetzung, Belastungsveränderung, Stationsauslastung, Personalausstattung, Wechsel Anordnungen, Verausgabungs-Belohnungsungleichgewichte, COVID-Infektionsangst, COVID-Risikogruppe, eigene Infektion, Erholung, Vertrauen in Kollegen, Sicherheitsgefühl<br>• IES<br>• PHQ	**Beanspruchung**<br>• PTBS/IES: M = 15,7 (Medizin: 14,8, MTA: 17,1)

Autoren, Zeitraum, Region	Studienziel, Methode, Stichprobenumfang	Messinstrumente	Hauptergebnisse
		• GAD<br>• Erschöpfung<br>• ENRICHD Social Support Inventory<br>• Schlaf, Alkohol, Rauchen	
[59],<br>4/2020,<br>Deutschland	• Stresserleben, gesundheitsbezogene Sorgen, Arbeitsengagement in Abhängigkeit des Kontaktes zu COVID-Patienten<br>• Zusammenhang Stresserleben, Arbeitsengagement<br>• Online-Survey<br>• N = 855	• Einzelitems zu Sorgen um Gesundheit, Stresserleben in der Pandemie<br>• UWES	**Belastung**<br>• 83% besorgt um Angehörigenwohl, 73% besorgt um Patienten, 39% besorgt um eigene Gesundheit; Sorge höher bei Kontakt mit COVID-19-Patienten<br>**Beanspruchung**<br>• 54% durch Pandemie im Arbeitsalltag gestresst<br>• Engagement 51% immer, sehr häufig/häufig von Arbeit begeistert, 49% immer, sehr häufig/häufig Aufgehen in Arbeit, 38% immer, sehr häufig/häufig engergiegeladen (Engagement)<br>• im Vergleich zu Normwerten geringes Engagement (Cut-off: 4,2)<br>**Zusammenhänge**<br>• bei Kontakt zu COVID-Erkrankten höhere Stresslevel, mehr Sorgen um Gesundheit der Angehörigen/Bekannten als ohne Kontakt<br>• pandemiebezogenes Stresserleben, Sorgen um eigene Gesundheit mit weniger Arbeitsengagement assoziiert, Sorge um Gesundheit der Patienten/Bewohner mit mehr Engagement assoziiert
[60],<br>7/2021,<br>Köln	• teamspezifische Belastungen<br>• Ressourcen/Entlastungsstrategien<br>• Positive/negative Erfahrungen, Wünsche<br>• Gruppendiskussion<br>• N = 22 Pflegekräfte		**Belastung**<br>• Emotionale Stressoren: z.B. schwere Krankheitsverläufe junger Patienten, häufige Todesfälle, verzweifelte Angehörige/Patienten, Sterbebegleitung, Angst vor eigener Ansteckung oder der Familie<br>• Körperliche Stressoren: z.B. Schutzkleidung, allein Aufgaben erfüllen die sonst zu zweit zu tun sind, adipöse Patienten<br>• Organisatorische Stressoren: z.B. Kommunikation, unterschiedliche Behandlungskonzepte, zusätzliche Aufgaben, Vielzahl gleichzeitig zu bewältigender Aufgaben, neue Prozeduren ohne Einarbeitung<br>**Ressourcen**<br>• Teamzusammenhalt, interprofessionelle Zusammenarbeit (Verlässlichkeit, Kooperation, kollegialer Austausch, Unterstützung, Humor, Solidarität), Sinnaspekt, Erfolgserlebnisse, Lernen, Wertschätzung

Autoren, Zeitraum, Region	Studienziel, Methode, Stichprobenumfang	Messinstrumente	Hauptergebnisse
[61], 3/2020-4/2020, Augsburg	• psychosoziale Beanspruchung in Abhängigkeit der Exposition mit COVID-19-Patient/innen und Vergleich Pflege/Medizin<br>• Prädiktoren von Beanspruchung<br>• Ressourcen/Entlastungsmöglich-keiten<br>• Online-Survey<br>• N = 75	• Offene Fragen zu arbeitsbezoge-nen Stressoren/psychosozialen Stressoren<br>• Angst vor COVID-Infektion<br>• PHQ (Subskalen Stresserleben, Angst, Depression)<br>• MBI (Burnout)<br>• Stresserleben aufgrund aktueller Situation<br>• offene Fragen Verbesserungsvorschlägen	**Belastung**<br>• 55% Covid-19-Bereich vs. 12,5% reguläre Stationen Zunahme Workload, Konflikte, Teamveränderungen<br>• 22,5% Covid-19-Bereich vs. 50% Belastung durch Zukunftssorgen<br>**Beanspruchung**<br>• Pflegepersonal auf COVID-19-Stationen mehr Stresserleben, depressive Symptome, Erschöpfung, weniger professionelle Effizienz<br>**Ressourcen**<br>• Freizeit, psychosoziale Unterstützung durch Familie/Freunde<br>• 62% des Pflegepersonals auf COVID-19-Stationen sieht Verbesserungsbe-darf für Organisation/Planung (Personalausstattung, stabile Teams, mehr Räume)

Anmerkung: BAM = Brief Agitation Measure, BAT = Burnout Assessment Tool, BHS = Beck Hopelessness Scale, BRS = Brief Resilience Scale, CoPaQ = COVID-19-Pandemic Mental Health Questionaire, COPSOQ = Copenhagen Psychosocial Questionnaire, DASS = Depression, anxiety and stress scales, DESC = Rasch-based depression screening, ERI = Efford Reward Imbalance Fragebogen, ESSI = Enrichd Social Support Inventory, GAD = Generalized Anxiety Disorder Scale-2, GTL = Global Transformational Leadership Scale, INQ = Interpersonal Needs Questionnaire, IwS = Index for Work Satisfaction, MSQ = Minnesota Satisfaction Questionaire, PANAS = Positive and Negative Affect Schedule, PHQ = Patients-Health-Questionnaire, PSA = persönli-che Schutzausrüstung, PSQI = Pittsburgh Sleep Quality Index, SDASS = Stress Scale der DASS, SDES = Short Defeat and Entrapment Scale, SSEV = Suicide Ideation and Behaviour Scale, SOC-Scale German ultashort version of the Sence-of-Coherence-Scale, UWES = Utrecht Work Engagement Scale, WAI = Work-Ability-Index
